# Improved annotation of the domestic pig genome through integration of Iso-Seq and RNA-seq data

**DOI:** 10.1186/s12864-019-5709-y

**Published:** 2019-05-07

**Authors:** H. Beiki, H. Liu, J. Huang, N. Manchanda, D. Nonneman, T. P. L. Smith, J. M. Reecy, C. K. Tuggle

**Affiliations:** 1 Department of Animal Science, Iowa State University, 2255 Kildee Hall, Ames, IA 50011 USA; 2 College of Animal Science and Technology, Jiangxi Agricultural University, Nanchang, Jiangxi People’s Republic of China; 3 Department of Ecology, Evolution, and Organismal Biology, Iowa State University, 819 Wallace Road, Ames, IA 50011 USA; 4 USDA, ARS, U.S. Meat Animal Research Center, Clay Center, NE 68933 USA

**Keywords:** Porcine, Transcriptome sequencing, PacBio, Iso-seq, Single molecule long read sequencing, RNA-seq, Genome annotation

## Abstract

**Background:**

Our understanding of the pig transcriptome is limited. RNA transcript diversity among nine tissues was assessed using poly(A) selected single-molecule long-read isoform sequencing (Iso-seq) and Illumina RNA sequencing (RNA-seq) from a single White cross-bred pig.

**Results:**

Across tissues, a total of 67,746 unique transcripts were observed, including 60.5% predicted protein-coding, 36.2% long non-coding RNA and 3.3% nonsense-mediated decay transcripts. On average, 90% of the splice junctions were supported by RNA-seq within tissue. A large proportion (80%) represented novel transcripts, mostly produced by known protein-coding genes (70%), while 17% corresponded to novel genes. On average, four transcripts per known gene (tpg) were identified; an increase over current EBI (1.9 tpg) and NCBI (2.9 tpg) annotations and closer to the number reported in human genome (4.2 tpg). Our new pig genome annotation extended more than 6000 known gene borders (5′ end extension, 3′ end extension, or both) compared to EBI or NCBI annotations. We validated a large proportion of these extensions by independent pig poly(A) selected 3′-RNA-seq data, or human FANTOM5 Cap Analysis of Gene Expression data. Further, we detected 10,465 novel genes (81% non-coding) not reported in current pig genome annotations. More than 80% of these novel genes had transcripts detected in > 1 tissue. In addition, more than 80% of novel intergenic genes with at least one transcript detected in liver tissue had H3K4me3 or H3K36me3 peaks mapping to their promoter and gene body, respectively, in independent liver chromatin immunoprecipitation data.

**Conclusions:**

These validated results show significant improvement over current pig genome annotations.

**Electronic supplementary material:**

The online version of this article (10.1186/s12864-019-5709-y) contains supplementary material, which is available to authorized users.

## Background

Domestic pigs (*Sus scrofa domesticus*) are closely related to humans in terms of anatomy, genetics and physiology and represent an excellent animal model in many fields of biomedical research [1, 2]. Comparative analysis indicates that there is more genetic similarity between pig and human than mouse and human [2]. In addition, experiments in pigs are much more likely to be predictive of therapeutic treatments in humans than experiments in rodents [2]. Despite the value of pigs to agriculture, food security and medicine, our current knowledge of pig genome functional elements is limited [3].

The recent, long read-based update to the pig reference genome assembly was a major step forward for swine research. This genome assembly (Sscrofa11.1) was annotated both at the European Bioinformatics Institute (EBI) [4] and National Center for Biotechnology Information (NCBI) [5]. Although these annotations represent significant improvement over the previous pig genome annotation (Sscrofa10.2) [6], they are still far from complete. For example, the number of annotated genes and transcripts per gene (tpg) in the current pig genome annotations (NCBI release 109: 30,334 genes and 2.9 tpg, Ensembl release 93: 25,880 genes and 1.9 tpg) are fewer than reported for genome of human (NCBI release 109: 54,644 genes and 4.2 tpg, Ensembl release 93: 57,373 genes and 3.5 tpg, coding, non-coding and pseudogenes were included in this calculation). The most significant difference is in the number of non-coding genes. Despite the fact that non-coding regions of the human genome harbor a rich array of functionally significant elements [7, 8] (e.g. majority of trait-associated loci in human genome located outside protein coding regions [9–11]), very few numbers of these elements have been annotated in the current pig genome annotations (NCBI release 109: 6460 non-coding genes, Ensembl release 93: 3250 non-coding genes) compared to the report in human genome (NCBI release 109: 17,835 non-coding genes, Ensembl release 93: 22,107 non-coding genes). The characteristics of NCBI and Ensembl genome annotations differ because of variations in their annotation strategies and resources [5, 12, 13]. Ensembl annotates using an automated process called Ensembl genebuild pipeline, whose main focus is to generate a conservative set of protein-coding gene models in combination with manual annotation provided by HAVANA team [12]. The Ensembl genebuild pipeline uses the following steps to annotate protein-coding genes [12]: (1) produce gene models using species-specific proteins and proteins from closely related species, species-specific cDNAs and Expressed Sequence Tags (ESTs), short-RNA sequencing and long-RNA sequencing data, (2) add Untranslated regions (UTRs) to derived gene models from previous step using species-specific cDNAs and ESTs and RNA sequencing data and (3) merge exon match transcripts built in the first step to produce multi-transcript genes. The NCBI pipeline [13] passes protein and RNA read alignments to Gnomon [5] for gene prediction and then integrates the results with available RefSeq transcripts to select the best gene models. As described by Thibaud et al. [13]:

“Gnomon first chains together non-conflicting alignments into putative models. In a second step, Gnomon extends predictions missing a start or a stop codon or internal exon(s) using an HMM-based algorithm. Gnomon additionally creates pure *ab initio* predictions where open reading frames of sufficient length but with no supporting alignment are detected”.

These are how the two annotations are different for coding regions, and we investigated these as well as other types of transcripts in this study.

Deciphering transcriptome (the total RNA molecules produced from the genes of an organism) complexity is critical to connect the genome sequence to gene function [14–16]. Next-generation sequencing (NGS) technologies (e.g. Illumina) that can produce millions of high quality (99% base-level accuracy) sequence reads was an important step towards the elucidation of tissue transcriptomes [17, 18]. However, the sequence read length of NGS technologies (100–150 base pairs, bp) is much shorter than the actual transcript lengths (the median length of human transcripts is about 2.5 k bases, kb). This creates computational challenges to accurately deciphering full-length transcripts [19–21]. In recent years single-molecule long-read isoform sequencing (Iso-seq) technology was developed [22] capable of producing reads > 4 kb, providing an alternative approach to overcome many of these limitations [23]. Indeed, Iso-seq data has been used for genome annotation of different species from Maize to Human [24–26]. However, the error rate in single molecule sequencing on the Pacific Biosciences (PacBio) platform (15–20%) is much higher than for the Illumina platform sequence reads (1%) [17]. In addition, the error model of both technologies differs. Although Illumina reads mainly contain miscalled bases with increasing frequency toward the end of sequence reads, PacBio generates primarily insertions (10%) and deletions (5%) in a random pattern [17]. The accuracy of PacBio long reads can be increased using in silico hybrid error correction approaches by Illumina reads from matched samples [17, 27].

A recent study on the pig transcriptome [28] used PacBio Iso-seq data from 38 porcine tissues to improve the previous pig genome assembly (Sscr10.2). However, this study pooled tissue RNAs together prior to library creation, which makes it impossible to trace transcripts back to the original tissue and study transcript variability among porcine tissues. To identify a more complete catalogue of transcript isoforms across porcine tissues, we processed poly(A) selected PacBio Iso-seq and Illumina RNA sequencing (RNA-seq) data from nine tissues (brain, hypothalamus, liver, muscle, thymus, pituitary, small intestine, spleen and diaphragm). This data provided evidence to improve the annotation of thousands of protein-coding and long non-coding RNA (lncRNA) genes, such that the complexity of the pig transcriptome (number of transcripts per gene, lncRNA transcripts and alternative splicing events) is similar to that reported for the highly-annotated human genome. We also provide direct evidence that the predicted novel genes and transcripts are valid for creating improved annotation, by performing independent chromatin immunoprecipitation sequencing (ChIP-seq), poly(A) selected 3′-RNA-seq experiment and human FANTOM5 CAP Analysis of Gene Expression (CAGE) data. We show that these complementary technologies directly support the validity of our additions to annotation of the pig reference genome.

## Results

### Transcript level analyses-transcript diversity across tissues

The extent of RNA transcript diversity among nine different porcine tissues (brain, diaphragm, hypothalamus, liver, longissimus dorsi (LD) muscle, pituitary, small intestine, spleen and thymus), collected from a healthy 48-day old crossbred barrow pig (Yorkshire $$ \left(\raisebox{1ex}{$5$}\!\left/ \!\raisebox{-1ex}{$8$}\right.\right) $$ x Landrace $$ \left(\raisebox{1ex}{$1$}\!\left/ \!\raisebox{-1ex}{$4$}\right.\right) $$ x Duroc $$ \left(\raisebox{1ex}{$1$}\!\left/ \!\raisebox{-1ex}{$8$}\right.\right) $$), was assessed using poly(A) selected PacBio Iso-seq (Additional file 1: Table S1) and Illumina RNA-seq data (Additional file 1: Table S2). A total of approximately 4.4 M Iso-seq reads and 499 M RNA-seq reads were collected, with a minimum of 398,629 (399 K) Iso-seq and 32,689,730 (32.7 M) RNA-seq reads from each tissue (average 491 K ± 92 K and 55 M ± 20 M, respectively) (Additional file 1: Table S1 and Additional file 1: Table S2). The RNA-seq data was not independently assembled; instead transcripts and transcript isoforms were defined from the Iso-seq reads and error-corrected, validated, and quantified using the short reads. This approach identified a total of 67,746 unique transcripts (1.2% of total Iso-seq reads) across all nine tissues. Predicted classification of these transcripts identified 41,003 (60.5%) predicted protein-coding, 24,527 (36.2%) lncRNA and 2216 (3.3%) non-sense mediated decay (NMD) transcripts (Additional file 1: Figure S1a). The error-corrected transcripts had a median length of 2900 nucleotides (nt; Additional file 1: Figure S1b), and mapped to the Sscrofa11.1 assembly to identify exons and introns. The median length of exons was 136 nt, and of introns was 1428 nt (Additional file 1: Figure S1b-d). On average, there were 6 exons per transcript (Additional file 1: Figure S1e) and most (97–98%) of the predicted acceptor and donor splice sites conformed to the canonical consensus sequences (Additional file 1: Figure S1f). An average of 90% of predicted splice junctions across the nine tissues were supported by Illumina-seq reads that spanned the splice junction (Additional file 1: Figure S2), supporting the accuracy of the transcript definition from Iso-seq reads.

**Fig. 1:**
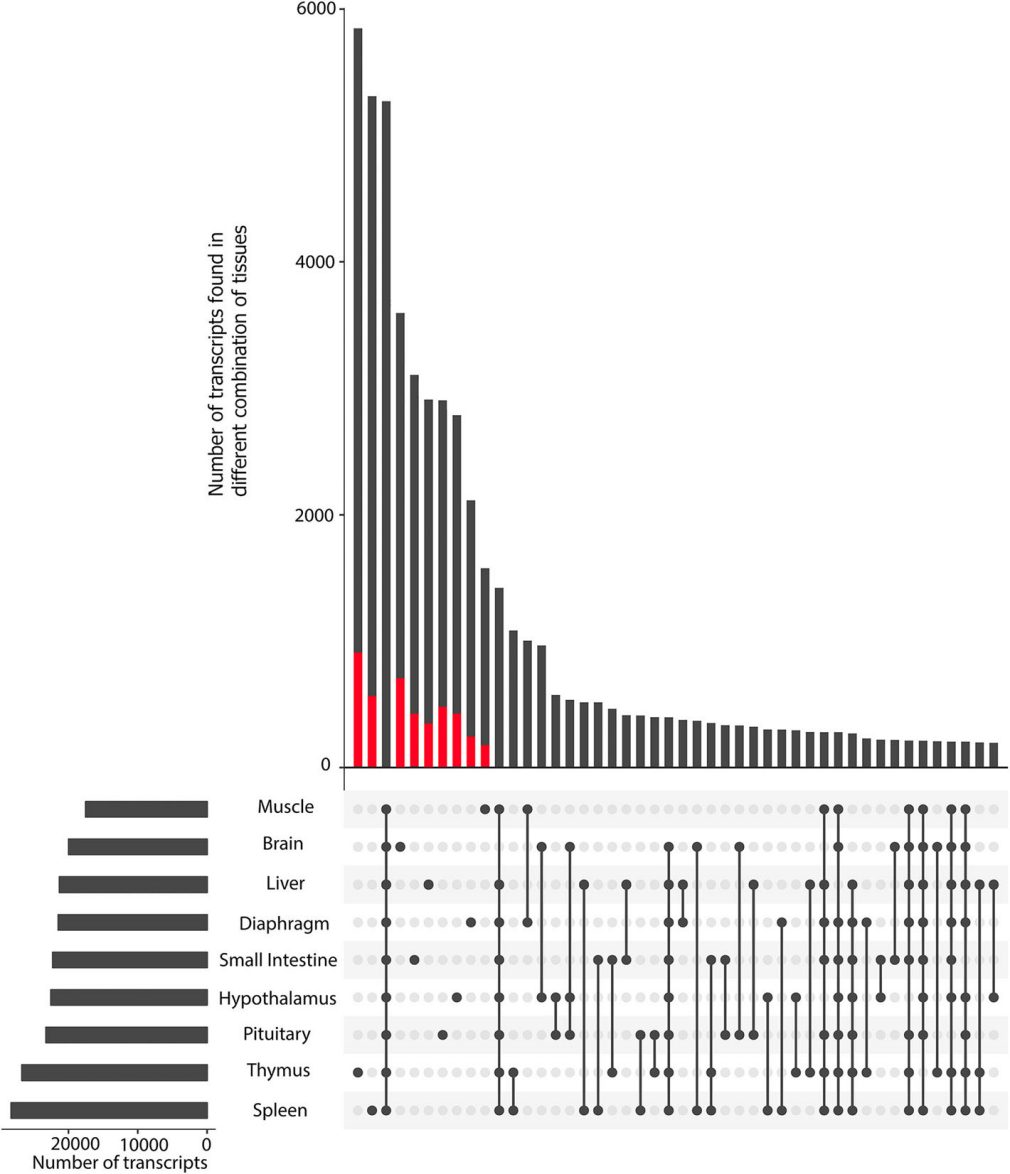
Number of detected transcripts in each tissue and their intersections with other tissues using UpSetR [65]. Red color identifies the proportion of tissue-specific (TS) transcripts that are produced by non-TS genes

We evaluated the set of Iso-seq-defined transcripts for potential tissue-specific transcripts. RNA-seq data were used to test whether the absence of these transcript from the Iso-seq reads in the other tissues is due to tissue-specificity or potentially due to lack of data. From the complete set of 4733 unique brain transcripts that were not observed in the Iso-seq data from any other tissue, 1136 (24%) transcripts had RNA-seq reads spanning all splice junctions in at least one other tissue, and these reads represent transcripts with expression levels more than 0.1 FPKM (inflection point in expression plot of transcripts detected in more than one tissue by Iso-seq data; Additional file 1: Figure S3) (see blue bars in Additional file 1: Figure S4). Thus, reliance on just Iso-seq data to predict tissue-specific transcripts may overestimate tissue-specificity due to a high false negative rate for transcript detection. To solve this over prediction of tissue specificity problem, we marked a transcript as “detected” in a given tissue only if (1) it had been detected by Iso-seq data in that tissue or (2) it had been detected by Iso-seq data in another tissue but all of its splice junctions were validated by Illumina reads in the tissue of interest with expression level more than 0.1 FPKM (see Methods section). This resulted in a total of 37,595 (55%) transcripts detected between 2 and 9 tissues and 30,151 tissue-specific transcripts (44%) (Fig. 1). While brain had lower numbers of detected transcripts (19,793) compared to other tissues (except muscle), it ranked third in terms of tissue specific transcripts (3597) (Fig. 1). Brain had the lowest number of transcripts per gene (1.65) compared to the other tissues. Furthermore, 25% of transcripts detected in the brain (4979) were from single-transcript genes, which was greater than any other tissues (Table 1). In addition, brain transcripts had on average one exon fewer (5 exons per transcript) than the other tissues (Table 1). Spleen had the highest number of detected transcripts (28,269) and 72% of these transcripts were produced by multi-transcript genes (averaging 2 transcripts per gene), which was more than that observed for the other tissues (Fig. 1). In general, groups of functionally-related tissues, such as thymus-spleen, muscle-diaphragm and hypothalamus-brain, tended to have more specific transcripts (detected only in these groups of tissues) than other pairwise tissue combinations (such as diaphragm-brain) (Fig. 1).

**Table 1:** Distribution of detected genes and transcripts across tissues

Tissue	Number of detected genes	Number of detected transcripts^1^	Average Number of exons per transcript	Number of transcripts produced by multi-transcript genes	Number of transcripts produced by single-transcript genes
Brain	12,064	19,973 (1.65)	5.20	14,994	4979
Diaphragm	11,417	21,468 (1.88)	6.22	17,453	4015
Hypothalamus	13,041	22,528 (1.72)	6.12	17,039	5489
Liver	11,118	22,008 (1.97)	6.33	18,028	3980
Muscle	9930	22,528 (2.26)	6.12	14,175	3334
Pituitary	12,662	23,240 (1.83)	6.26	18,098	5142
Small intestine	11,906	22,268 (1.87)	6.38	17,798	4470
Spleen	13,604	28,269 (2.07)	6.40	22,789	5480
Thymus	12,895	26,721 (2.07)	6.44	21,522	5199

^1^Number in parenthesis shows the number of transcripts per gene

#### Comparison of transcript structures across current pig genome annotations

**Fig. 2:**
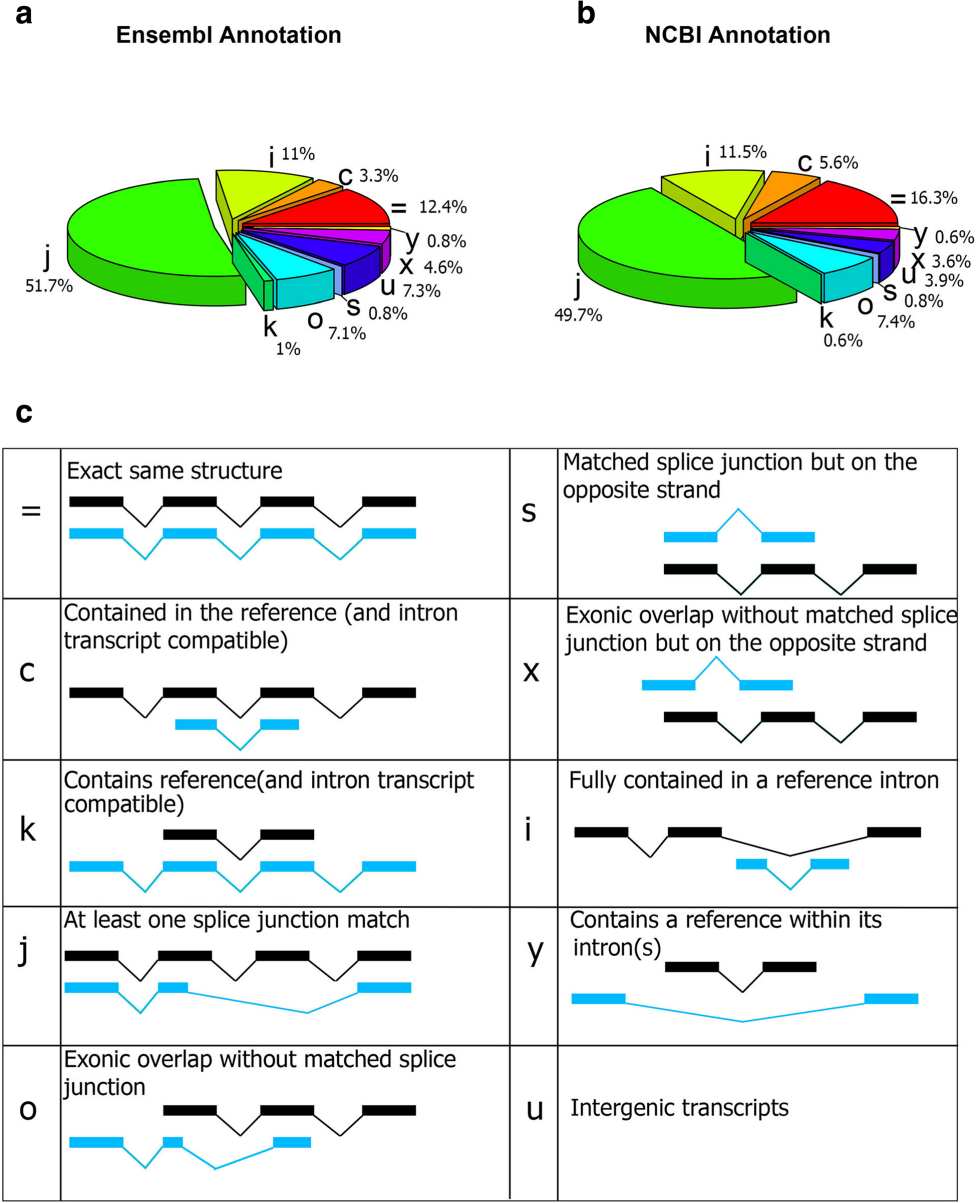
Comparision of PacBio transcript structure with known transcripts in Ensembl (**a**) and NCBI (**b**) genome annotations. (**c**) Exploratory key to different comparisons. Reference and predicted Iso-seq transcripts are identified by black and blue color, respectively

**Fig. 3:**
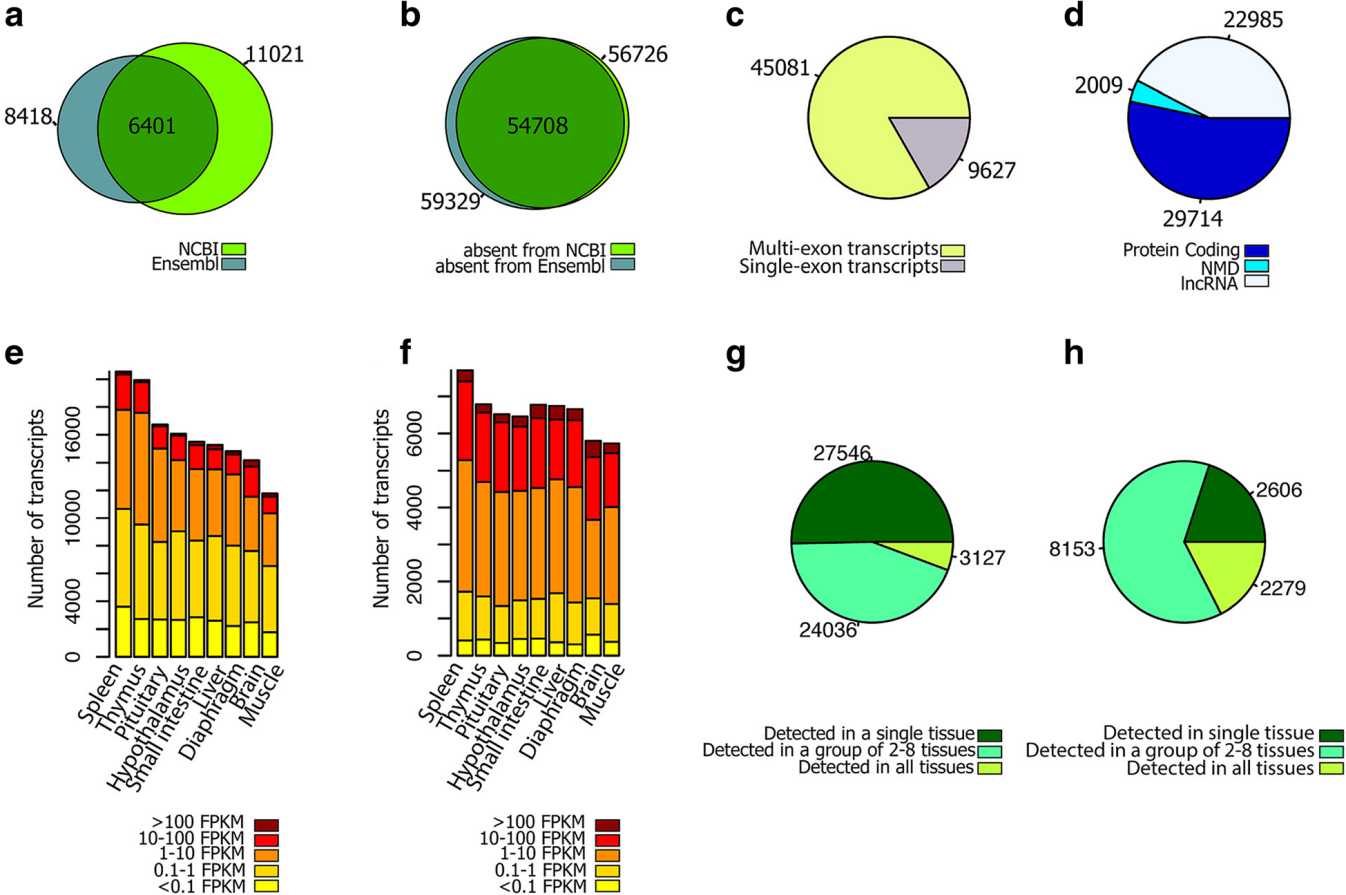
Venn diagram of known (**a**) and novel (**b**) PacBio transcripts based on Ensembl and NCBI annotations. (**c**) Classification of PacBio transcripts to spliced and non-spliced transcripts. (**d**) Novel transcripts biotypes. Expression level of known (**e**) and novel (**f**) transcripts across different tissues. Classification of known (**g**) and novel (**h**) transcripts based on the number of tissues in which they were detected

Comparing predicted transcript isoforms with known transcripts in the current pig genome annotations (Ensembl release 93 and NCBI Release 109) resulted in a total of 13,038 annotated transcripts exactly matching previously annotated transcripts (19% of all transcripts, or class “=” transcripts in Fig. 2), including 11,021 annotated NCBI transcripts, 8418 annotated Ensembl transcripts and 6401 transcripts that were common to both annotation gene sets (Fig. 2 and Fig. 3a). The remaining 54,708 transcripts (81%) in the Iso-seq data had no counterpart in currently available porcine genome annotations (Ensembl and NCBI), which we denote as predicted novel transcripts (Fig. 3b). A majority of these transcripts were spliced (82%; Fig. 3c) and protein-coding (54%; Fig. 3d). In general, these novel transcripts had a lower expression level as compared to known transcripts (Fig. 3e, f), and 50% of them were only detected in a single tissue (Fig. 3g). This proportion was 20% for known transcripts (Fig. 3h).

To study these novel transcripts in more detail, we classified them based on their structural similarity with annotated transcripts from Ensembl or NCBI annotations, into 9 different classes. Class “j” included transcripts that had at least a one splice junction in common with a reference transcript (Fig. 2). This class contained more than 60% of novel transcripts in either Ensembl-referenced transcripts or NCBI-referenced transcripts (Fig. 2). Class “k” included transcripts that extended the reference transcript (Fig. 2). This class included 815 Ensembl-referenced transcripts and 492 NCBI-referenced transcripts (Additional file 1: Figure S5a). There were 105 transcripts with “class k” that were present in both of the Ensembl and NCBI gene sets (Additional file 1: Figure S5a), which included 58 protein-coding, 40 lncRNA and 7 NMD transcripts (see Methods for NMD definition) (Additional file 1: Figure S5b). When averaged across tissues, 60% of class “k” transcripts had an expression level greater than 1 FPKM in their detected tissue (Additional file 1: Figure S5c). In addition, around 60% of these transcripts were detected in more than one tissue (Additional file 1: Figure S5d). These transcripts could be potentially used as the reference transcript in current *Sus scrofa* annotations. Class code “c” included transcripts that were contained in a reference transcript (Fig. 2). This class included 3% (2260 transcripts) of Ensembl-referenced transcripts and 5% (3801 transcripts) of NCBI-referenced transcripts (Fig. 2). On average, 75% of the transcripts included in “=”, “j”, “c” and “k” classes were protein-coding (Additional file 1: Figure S6). Class “o” included transcripts that had an exon overlap, but no shared splice junction with a reference transcript (Fig. 2). Transcripts in this class comprised 3% (2260 transcripts) of Ensembl-referenced transcripts and 7% (3801 transcripts) of NCBI-referenced transcripts. Class “s”, included transcripts that contained at least one shared splice junction with their reference transcripts, but on the opposite strand of the genome (Fig. 2). There were 448 transcripts with this structure in both Ensembl-referenced and NCBI-referenced transcripts and more that 90% of them (417 transcripts) were detected in more than one tissue (Additional file 1: Figure S7). Class “x” included transcripts with the same structure as class “o”, but on the opposite DNA strand (Fig. 2). There were 1662 transcripts with this structure in both Ensemb and NCBI annotations and 60% (997) of them were detected in more than one tissue. Classes “i”, “y” and “u” included transcripts that did not overlap with Ensembl or NCBI transcripts (Fig. 2). On average, 74% of class “i”, “y” and “u” transcripts were lncRNA (Additional file 1: Figure S6).

### Gene level analyses

**Fig. 4:**
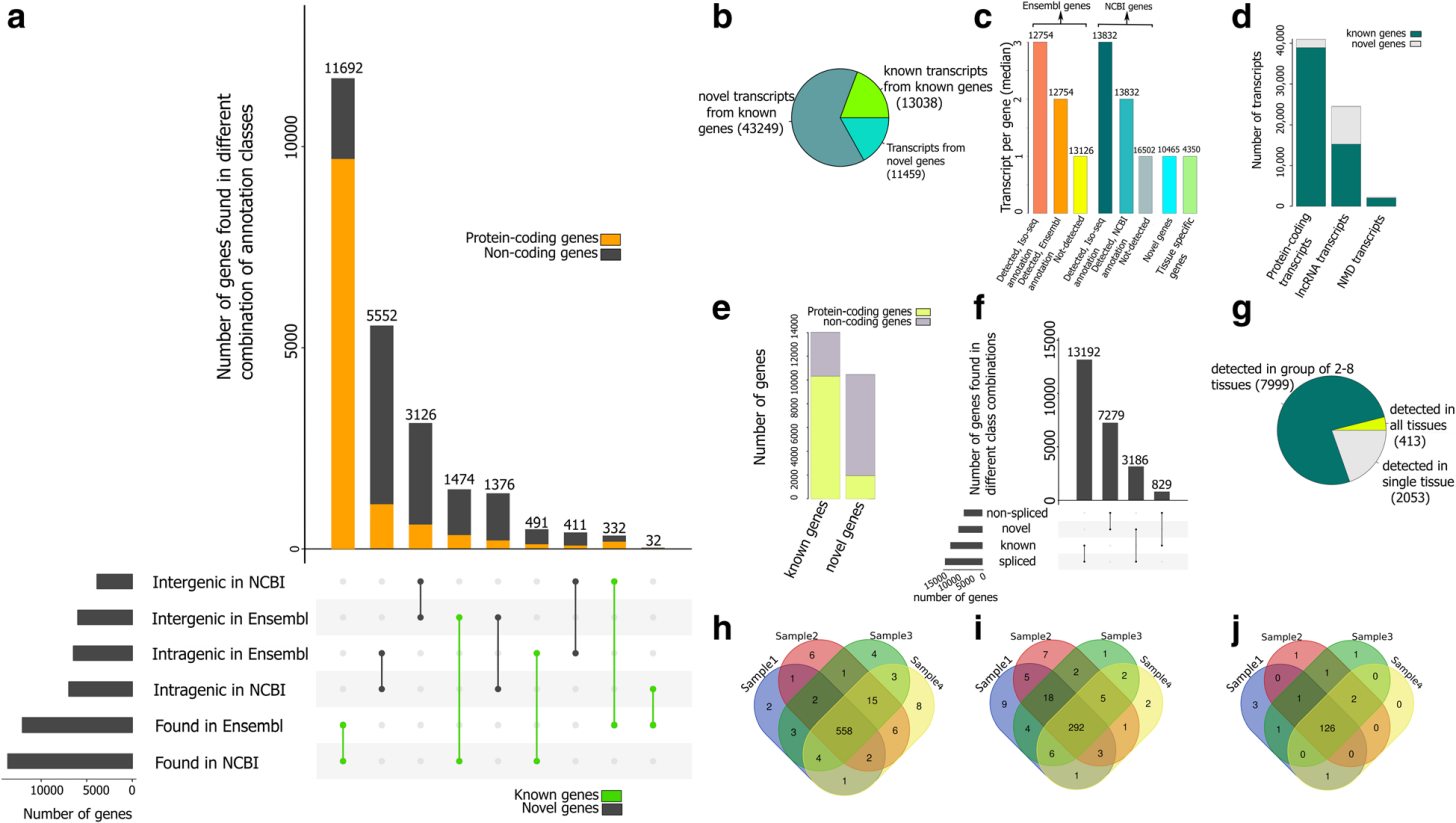
(**a**) Classification of predicted Iso-seq genes into known, novel-intergenic and novel-intragenic genes using Ensembl (release93) and NCBI (release 109) Sscrofa11.1 annotations by UpSetR [65]. Proportion of protein-coding genes in each class is identified by “orange” color. Intersections related to annotated genes are identified by “green” lines. (**b**) Distribution of transcripts across different classes of predicted genes. (**c**) Comparison of predicted and annotated genes in term of average number of produced transcripts. Number of genes in each class is shown on each bar. (**d**) Proportion of transcripts produced by novel and known genes in different transcript biotypes. (**e**) Gene biotypes. (**f**) Classification of genes into spliced and un-spliced genes using UpSetR [65]. (**g**) Classification of novel genes based on the number of tissues in which they were detected. (**h**) Validation of novel-intergenic genes detected in liver tissue by an independent liver chromatin immunoprecipitation (ChIP) sequencing experiment (2 histone modifications per sample). Venn diagram shows the distribution of 616 validated genes (with significant H3K4m3e and H3K36me3 peaks) across samples. (**i**) validation of NCBI specific Iso-seq genes that were located in intergenic region of pig genome based on Ensembl gene set (see text) detected in liver tissue by an independent liver ChIP sequencing experiment (2 histone modifications per sample). Venn diagram shows the distribution of 358 validate genes (with significant H3K4m3e and H3K36me3 peaks) across samples. (**j**) validation of liver detected Ensembl specific Iso-seq genes that were located in intergenic region of pig genome based on Ensembl gene set (see text) by an independent liver ChIP sequencing experiment (2 histone modifications per sample). Venn diagram shows the distribution of 137 validate genes (with significant H3K4m3e and H3K36me3 peaks) across samples

Transcript that contained an exon that overlapped (“=”, “j”, “c”, “k” and “o”) with either an Ensembl or NCBI annotated gene was considered to belong to a known gene. This resulted in the identification of transcripts for 14,021 known genes or 57% of all Iso-seq data-associated genes (24,486) (Fig. 4a lower). Approximately 80% of novel transcripts (43,249) were associated with known genes (Fig. 4b). The median number of transcripts per known gene (tpg) was three, which was higher than that was observed in either the Ensembl (2 tpg) or NCBI (2 tpg) annotated gene sets (Fig. 4c). Known genes (in either Ensembl or NCBI gene sets) that we did not detect in our Iso-seq data (Fig. 4c) had only 1 tpg. This may indicate that these genes are likely to be lowly expressed or tissue-specific, i.e. expressed in tissues not represented here (Fig. 4c). Known genes were associated with 95% (38,956) of protein-coding transcripts, 62% (15,290) of lncRNA transcripts and more than 92% (2041) of NMD transcripts (Fig. 4d).

Further, we identified novel genes; i.e., predicted Iso-seq genes (see Method) that produced “s”, “x”, “i”, “y” and “u” transcript structures (Fig. 2) that were not found in either Ensembl and NCBI gene sets. This resulted in total of 10,465 novel genes or 43% of all Iso-seq data-associated genes. Novel genes were further classified into novel intragenic genes (with at least one “i” transcript, Fig. 2c) and novel intergenic genes (without “i” transcript). A total of 8678 novel genes had the same classification (5552 intergenic novel genes and 3126 intragenic novel genes) in both the Ensembl and NCBI gene sets (Fig. 4a). In contrast, 1787 novel genes had different classifications in the Ensembl (1376 intragenic novel genes and 411 intergenic novel genes) and NCBI gene sets (411 intragenic novel genes and 1376 intergenic novel genes) (Fig. 4a). Only 21% of the novel transcripts (11,459 out of 54,708) were associated with novel genes (Fig. 4b). These genes had fewer transcript per gene (1 tpg) than known genes (3 tpg) (Fig. 4c). Novel genes produced approximately 5% of the protein-coding transcripts (2047), 38% of the lncRNA transcripts (9237) and 8% of the NMD transcripts (175) (Fig. 4d). In addition, the proportion of protein-coding genes, i.e. genes that had at least one protein-coding transcript, was lower in novel genes (19% - 1950 genes) than in known genes (77% of annotated genes or 10,328 genes) (Fig. 4e). While the proportion of genes with spliced transcripts was 30% in novel genes (3186), it was 94% in known genes (13,192) (Fig. 4f).

We also investigated differences between the annotated gene sets. All possible combinations of presence or absence in NCBI and Ensembl annotations for the Iso-seq annotated genes, as well as intragenic/intergenic location relative to those annotations, were determined and are summarized in Fig. 4a. For example, there were 1965 Iso-seq genes that were found in the NCBI gene set but not in the Ensembl annotation gene set (NCBI specific Iso-seq genes). These genes were located in intergenic (1474 genes) or intragenic (491 genes) regions of pig genome based on Ensembl gene set (Fig. 4a, fourth and sixth bars). In contrast, only 364 Iso-seq genes were found in the Ensembl gene set, by not in the NCBI gene set (Ensembl specific Iso-seq genes). These genes were located in intergenic (332 genes) or intergenic (32 genes) regions of pig genome based on NCBI gene set. (Fig. 4a, last two bars). However, the proportion of protein-coding genes, i.e. genes that had at least one protein-coding transcript in these 364 Ensembl specific Iso-seq genes (56%) was higher than that for the 1965 NCBI specific Iso-seq genes (24%) (Fig. 4a).

#### Validation of novel genes

**Fig. 5:**
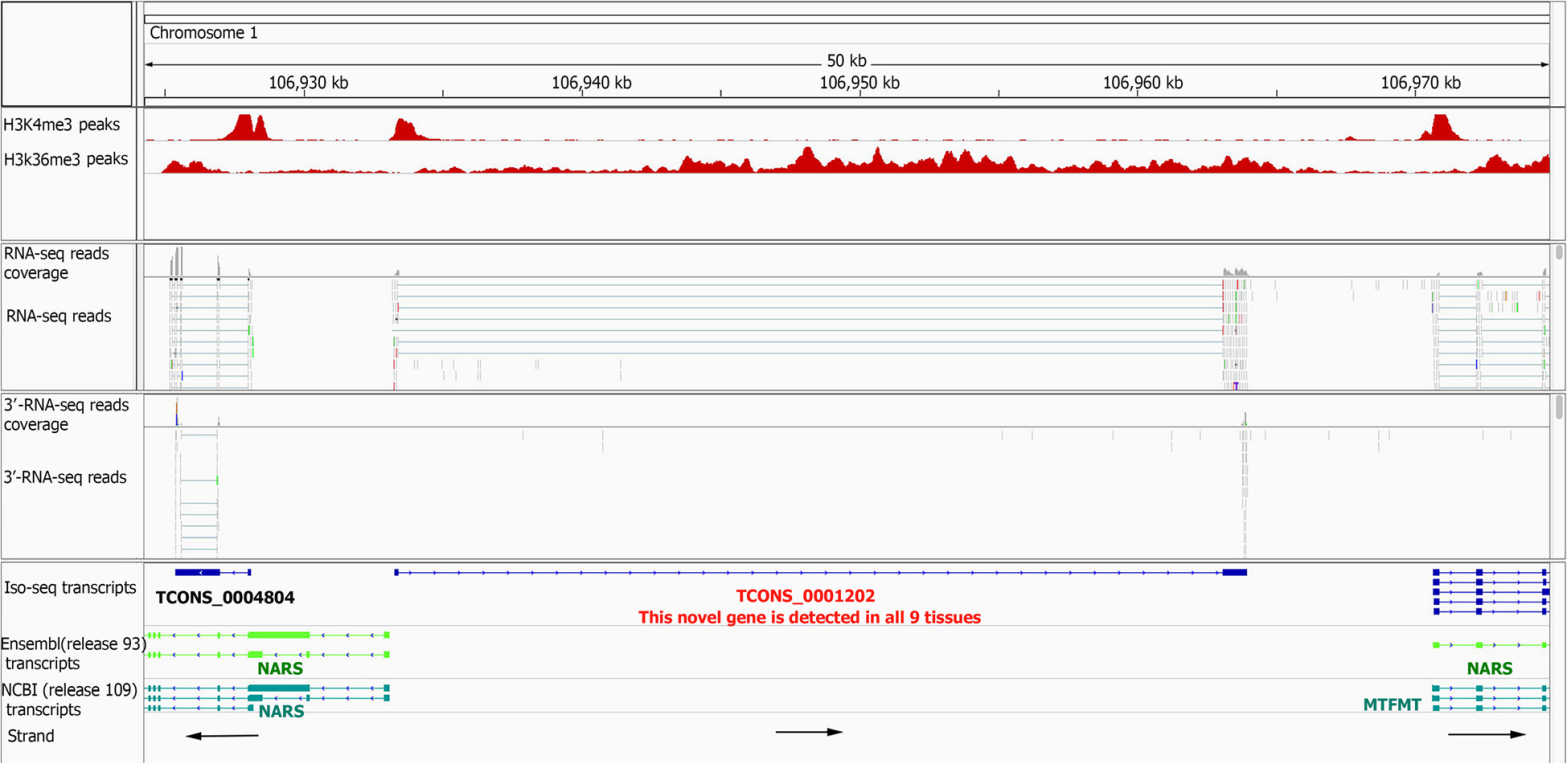
Example of validation of novel intergenic Iso-seq gene using matched RNA-seq reads and independent liver ChIP-seq (H3K4me3 and H3K36me3) and 3′-RNA-seq experiments

More than 80% of the novel genes had transcripts detected in more than one tissue (Fig. 4g). Interestingly, 413 novel genes had transcripts that were detected in all 9 tissues (Fig. 4g). Using data from an independent liver chromatin immunoprecipitation (ChIP) sequencing experiment (Additional file 1: Table S3), we found that more than 80% (616) of the novel Ensembl and NCBI intergenic genes detected in liver tissue (694) had significant H3K4me3 (tri-methylation of lysine 4 on histone H3) that mapped to their promoters, i.e. the genomic region that spans from 500 base pairs (bp) 5′ to 100 bp 3′ of the genes first exon (Fig. 4h, see illustrative examples in Fig. 5 and Additional file 1: Figure S8). Similar results were found for ChIP data using H3K36me3 (tri-methylation of lysine 36 on histone H3) peaks mapping to gene bodies (Fig. 5 and S8). In addition, around 80% of these genes (558 out of 616), had significant H3K4me3 and H3K36me3 peaks in all ChIP-seq samples (2 histone modifications per sample).

Out of 493 liver detected NCBI specific Iso-seq genes that were located in intergenic region of pig genome based on Ensembl gene set, 358 genes (72%) had H3K4me3 and H3K36me3 peaks that mapped to their promoter and gene body, respectively (Fig. 4i). This proportion was 82% (137 genes) for liver detected Ensembl specific Iso-seq genes that were located in intergenic region of pig genome based on NCBI gene set (165 genes) (Fig. 4j).

#### Identification and validation of annotated gene border extensions

**Fig. 6:**
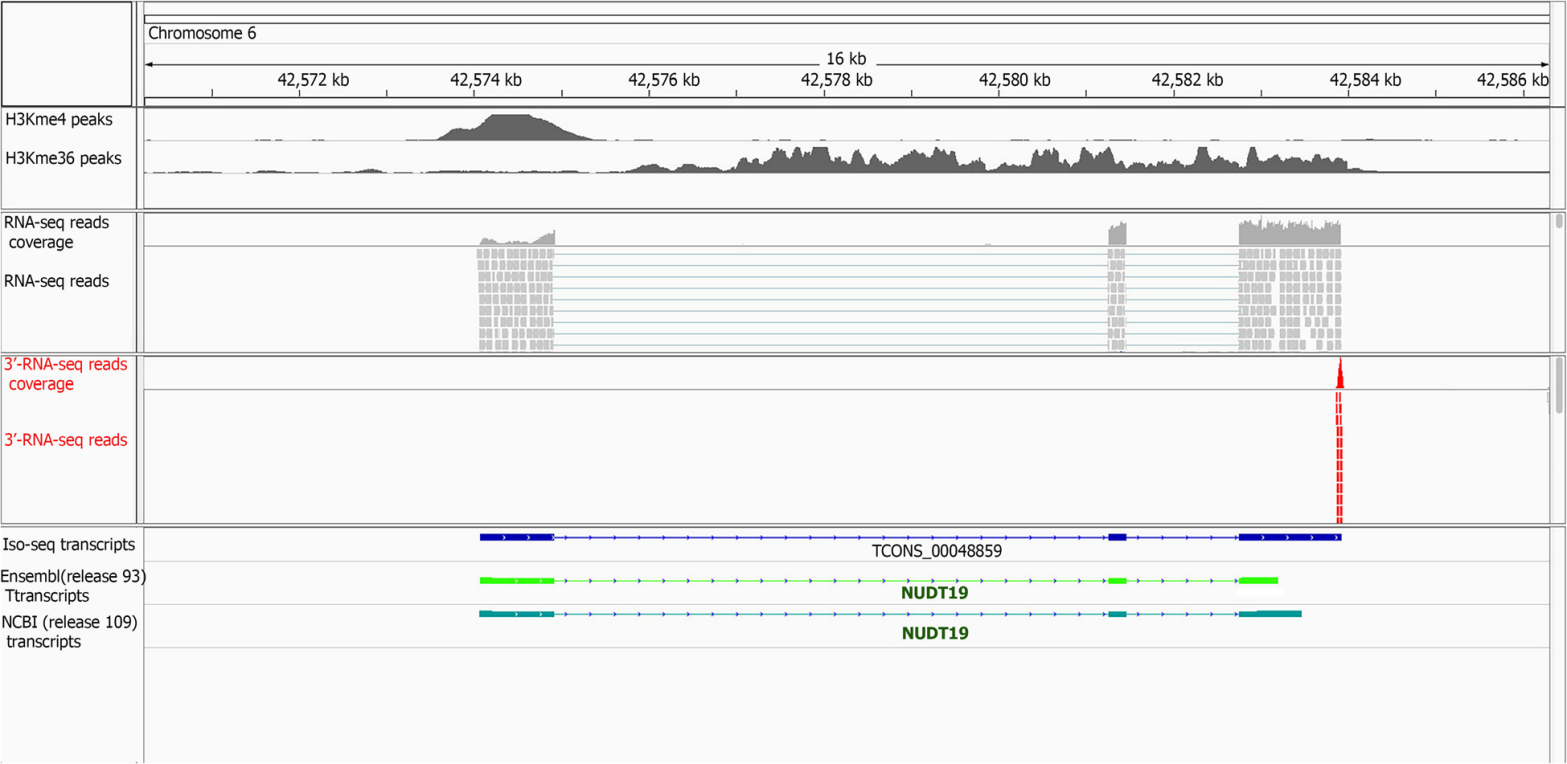
Example of validation of extended 3′ annotation using an independent liver 3′-RNA-seq experiment

This new Iso-seq based pig gene set annotation extended (5′ end extension, 3′ end extension or both) more than 6000 known Ensembl or NCBI gene borders (Table 2). Extensions were longer on the 3′ side, but the median increase was 90 nt for the latter group. To validate 3′ end extensions, an independent liver poly(A) selected 3′-RNA-seq dataset (Quantseq, Lexogen; Additional file 1: Table S4) was utilized. Out of 3228 3′ end extended Ensembl genes with transcripts detected in liver, 2902 genes (90%) had 3′-RNA-seq reads that mapped to the 3′ extension (Additional file 1: Figure S9a, and see illustrative examples in Fig. 6 and Additional file 1: Figure S10). Similarly, 88% of liver-detected 3′ end extended NCBI genes (2980 out of 3368) (Additional file 1: Figure S9b) were validated with this gene expression set. To measure the effect of these 3′ end extension events on gene expression values, we narrowed down the analysis to those liver detected Iso-seq genes with exact same 5′ end but extended 3′ end compared to the reference Ensembl genes (233 genes). The results showed that the expression level of the extended genes (read counts) increased on average 40% in Iso-seq genes compared to their matched Ensembl genes (Additional file 1: Figure S11).

**Table 2:** Gene border extensions in current Ssc11.1 genome annotations by PacBio Iso-seq data

Annotation	Type of gene extension	Number of genes	Median extension (nucleotides)
Ensembl (release93)	5′ extension only	1476	60
	3′ extension only	3160	395
	Both ends extended	1712	90 5’
			562 3’
NCBI (Release 109)	5′ extension only	1625	46
	3′ extension only	3560	550
	Both ends extended	1425	67 5’
			507 3’

**Fig. 7:**
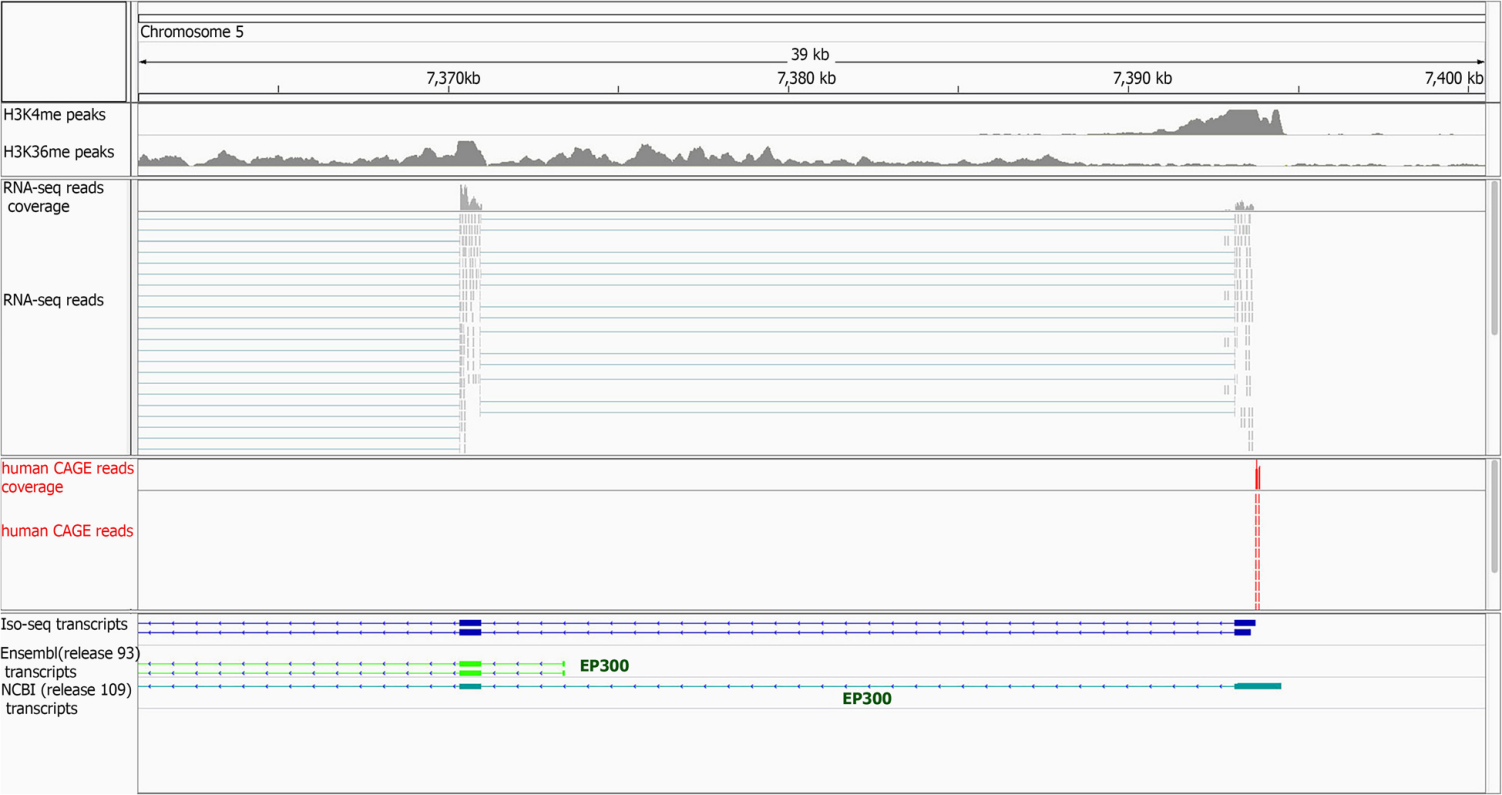
Example of validation of extended 5′ annotation using an independent Human CAGE data

To validate the 5′ end extension events, we used a total of 45,067,042 CAGE sequences from eight matched human tissues (brain, diaphragm, liver, LD muscle, pituitary, small intestine, spleen and thymus). Around 70% of the CAGE reads (31,486,934) mapped to the pig genome and 22% of them (6,963,037) were mapped uniquely to the genome. These uniquely mapped reads were used in the rest of the analyses. A total of 1270 human-pig orthologous genes, i.e. one-to-one orthologous genes with more than 90% nucleotide similarity [29], with an extended 5′ end based on Iso-seq data were selected for validation. The median genomic length of the extended 5′ end for these genes was 135 bp. The promoter region as defined by the median length of H3K4me3 peaks (600 bp) that overlapped with both the Iso-seq and Ensembl gene set annotations, is too broad to identify the correct 5′ end. To differentiate the Ensembl and Iso-seq defined 5′ ends, we developed an ad hoc method as described here. The candidate 5′ end region predicted by Ensembl or Iso-seq genes was defined based on the gene start site plus or minus 1/3 of the Ensembl gene extended region length (Additional file 1: Figure S12a). This allowed us to determine whether the human CAGE data (median length = 32 nt) mapped unambiguously to either the predicted extended exon, the Ensembl annotated 5′ end, or to neither (Additional file 1: Figure S12a). Out of 1270 human-pig orthologous genes with an extended 5′ end, 320 genes had human CAGE reads that uniquely mapped from the region defined as the Iso-seq candidate 5′ end to the Ensembl candidate 5′ end (Additional file 1: Figure S12a). This 320 gene subset was used to determine the validity of a gene’s 5′ end annotation from Ensembl or our analysis. Of these 320 genes, 203 genes (63%), had CAGE reads that mapped to the Iso-seq candidate 5′ end, i.e. these reads validated the Iso-seq 5′ end (Additional file 1: Figure S12a). This includes 105 genes with only validated Iso-seq 5′ end and 98 genes with both validated Iso-seq and Ensembl 5′ end (multiple promoter genes) (Fig. 7, Additional file 1: Figure S12b and Figure S13).

### Alternative splicing events

**Fig. 8:**
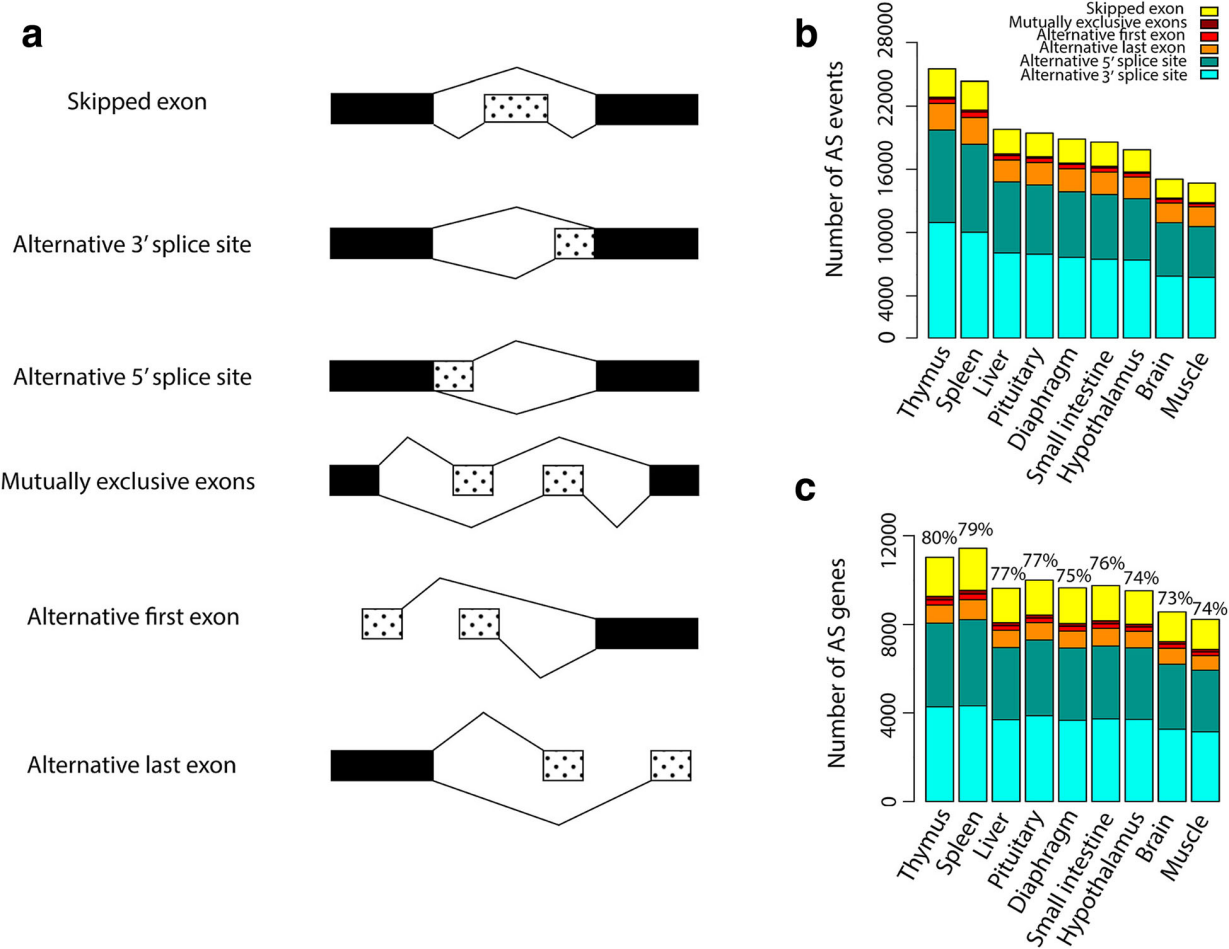
Different types of alternative splicing events and their variations within (**a**) and across (**b**) tissues. (**c**) Distribution of genes containing alternative splicing events within and across tissues. Numbers at the top of each bar showed the percentage of alternative event candidate genes (genes with at least 2 spliced transcripts) exhibiting one or more form of alternative splicing events

Alternative splicing events shown in Fig. 8a were classified into the seven major types as defined by [30]:

“These events are commonly distinguished in terms of whether RNA transcripts differ by inclusion or exclusion of an exon, in which case the exon involved is referred to as a ‘skipped exon’ (SE) or ‘cassette exon’, ‘alternative first exon’, ‘alternative last exon’. Alternatively spliced transcripts may also differ in the usage of a 5' splice site or 3' splice site, giving rise to alternative 5' splice site exons (A5Es) or alternative 3' splice site exons (A3Es), respectively. A sixth type of alternative splicing, ‘mutually exclusive exons’, in which one of two exons is retained in RNA but not both. These descriptions are not necessarily mutually exclusive; for example, an exon can have both an alternative 5' splice site and an alternative 3' splice site, or have an alternative 5' splice site or 3' splice site but be skipped in other transcripts. A seventh type of alternative splicing, ‘intron retention’, in which two transcripts differ by the presence of an unspliced intron in one transcript that is absent in the other”.

Transcripts showing this latter event were excluded from the analysis as it was difficult to distinguish true intron retention events from pre-RNA sequences. The proportion of alternative splicing events were uniform across the 9 tissues, and alternative 3′ splice site exons were the predominant splicing event followed by alternative 5′ splice site exons and skipped exons (Fig. 8b). Thymus had the highest number of alternative splicing events (Fig. 8b) with 80% of the AS event candidate genes (genes with at least 2 spliced transcripts) exhibiting one or more form of AS events (average of 4.4 AS events per gene), followed by spleen and pituitary (Fig. 8b, c). Brain and muscle had the lowest number of AS genes in this study (Fig. 8c).

### Tissue specific transcripts

**Fig. 9:**
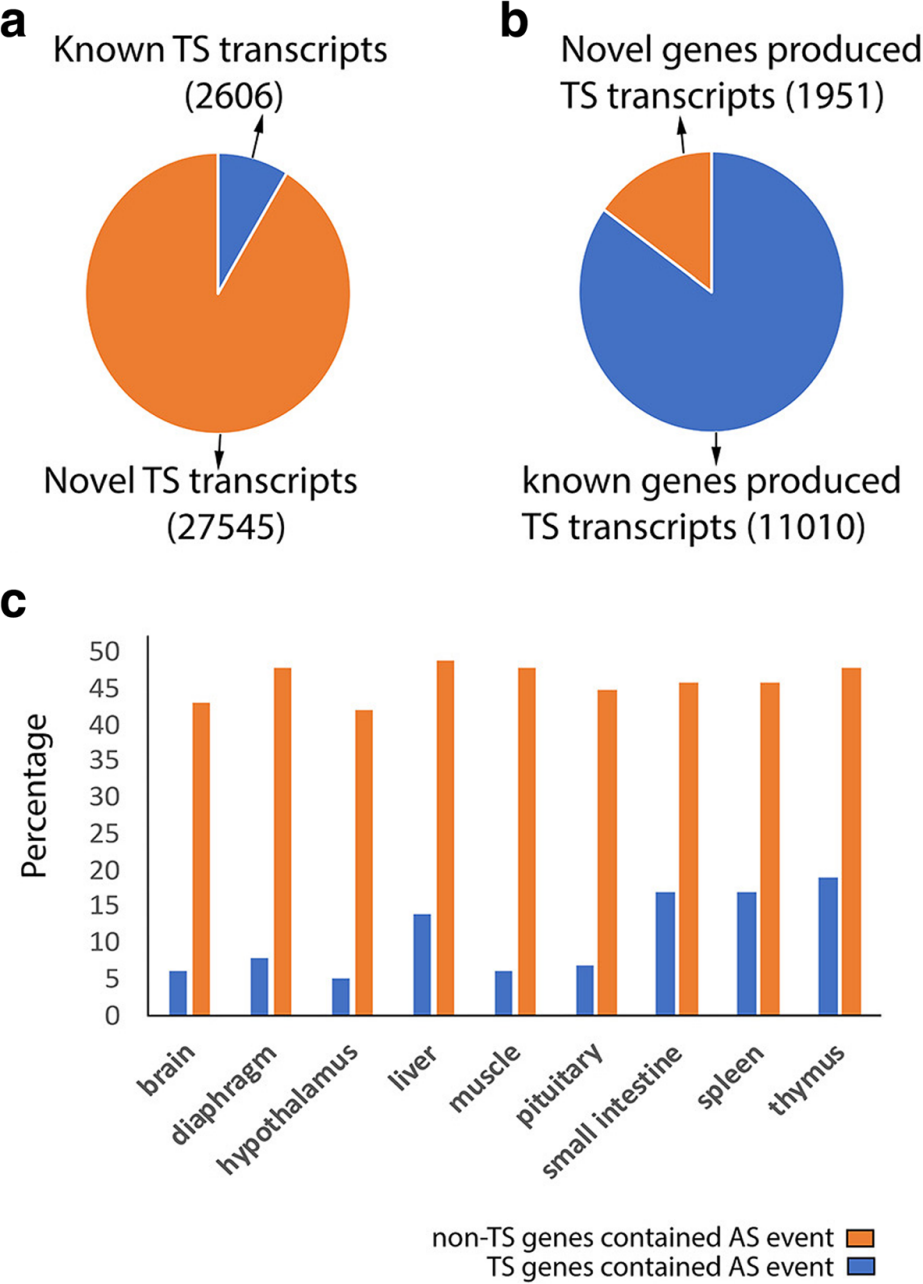
(**a**) Classification of tissue-specific (TS) transcripts based on their novelty. (**b**) Fraction of known and novel genes that produce at least a single TS transcript. (**c**) Proportion of TS genes and non-TS genes containing alternative splicing events

Forty-four percent of all transcripts (30,151) were only detected in a single tissue and were denoted as tissue-specific transcripts (Fig. 1). The proportion of tissue-specific transcripts was higher in novel transcripts than known transcripts (Fig. 3g and Fig. 3h) such that more than 90% of the tissue-specific transcripts (91%) represented novel transcripts (Fig. 9a). Also, a majority of tissue-specific transcripts were produced by known genes (Fig. 9b). Of 9 tissues, thymus had the highest proportion of tissue-specific transcripts (5847; 21%), followed by spleen (5309; 19%) and brain (3597; 18%); whereas muscle had the lowest (1576; 7%) (Fig. 1). Averaging across tissues, 17% of tissue-specific transcripts were produced by tissue specific genes and this proportion was highest in brain (24%) and lowest in diaphragm (12%) (red bars in Fig. 1). There was close concordance between enriched Gene Ontology (GO) terms in tissue-specific genes and the biological function of their related tissue (in Additional file 1: Table S5 we listed the top three enriched GO terms for each tissue-specific gene list). Alternative splicing events tended to be more prevalent in non-tissue specific genes than tissue specific genes (Fig. 9c).

### Unusual transcripts

**Fig. 10:**
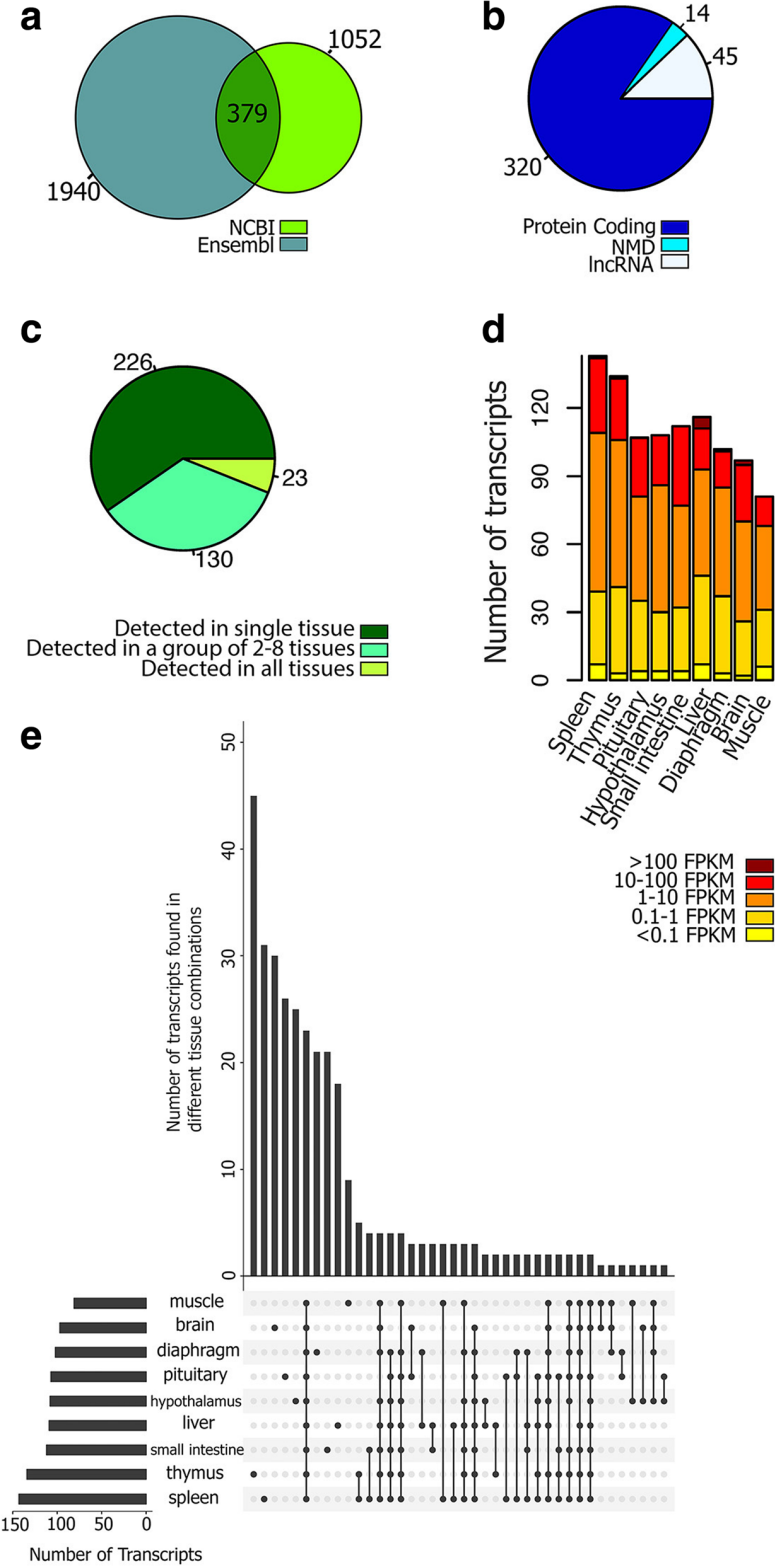
(**a**) Distribution of transcripts covering more than one known gene across Ensembl and NCBI annotations. (**b**) biotypes of transcripts with these structure in both Ensembl and NCBI annotations, their classification based on the number of detected tissues (**c**), their expression level in different tissues (**d**) and the number of transcripts detected in each tissue and their intersection with other tissues (**e**) using UpSetR [65]

**Fig. 11:**
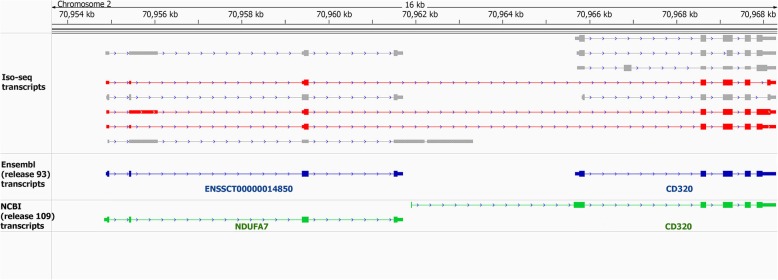
Example of transcripts covering multiple known genes (identified by red color). Predicted protein-coding region in each transcript is identified by thicker lines (see Methods for prediction of coding transcripts)

Three hundred and seventy-nine transcripts had an exon that overlapped with more than one Ensembl or NCBI annotated gene, which are referred to as fused-transcripts (Fig. 10a and Fig. 11). More than 80% of these transcripts (320) were protein-coding (Fig. 9b) and 40% of them were detected in more than 1 tissue (Fig. 10c). Averaging across tissues, 70% of fused-transcripts had an expression level more than one FPKM (Fig. 10d). Thymus had the highest number of tissue-specific-fused-transcripts (45) followed by spleen (31) and brain (30). In contrast, muscle had the lowest (9) number of fused transcripts (Fig. 10e). In addition, this group of transcripts had on average 9 exons, which was 2 exons more than other transcripts and they were more frequent in spleen (37% of them detected in this tissue) than other tissues.

## Discussion

Despite a lot of improvement in current pig genome annotation of Sscrofa11.1 assembly (Ensembl release 93 and NCBI release 109) compared to the previous genome assembly (Sscrofa10.2), these annotations are still far from complete. For example, in Ensembl (release 93) the number of annotated genes for the pig genome (25,880 genes) is around half of what has been reported in human (57,373 genes) as a closely related species [4]. In this study, using Iso-seq data from nine different porcine tissues, we could identify 10,465 novel genes not reported in current pig genome annotations (Ensembl release 93 and NCBI release 109). In addition, there were 1961 predicted Iso-seq genes reported in NCBI annotation but not in Ensembl annotation and 364 predicted Iso-seq genes reported in Ensembl annotation but not in NCBI annotation. The high frequency of validation of these novel genes using an independent liver chromatin immunoprecipitation sequencing experiment verifies the improvement of current pig annotations in term of the number of genes using our methods.

lncRNAs are important regulators of gene expression, and they are involved in a wide range of biological processes [31]. There are 9292 and 483 transcripts annotated as lncRNA in NCBI (Release 109) and Ensembl (release 93) pig annotation of Sscrofa11.1 assembly. In the human genome, this number is 25,786 (Ensembl release 93) that is similar to our 24,527 predicted lncRNA transcripts. A similar number of lncRNAs (20,516) have been reported in the chicken genome using Iso-seq data [24]. These numbers are likely an underestimation as the Iso-seq transcripts used in this study were poly(A) selected and we did not have the ability to capture non-polyadenylated lncRNAs. Also, during the process, we removed predicted single-exon transcripts that had only been detected in a single tissue by Iso-seq data as we could not verify whether they were real transcripts or fragments resulting from a decayed transcript. The selected single-exon transcripts had high base coverage with RNA-seq reads (on average, each base of single-exon transcripts was covered at least 16 times in the detected tissue by RNA-seq) implying that they are less likely to be genomic DNA contamination. This analysis produced a rate of 68% spliced transcripts in lncRNAs. However, the majority of spliced lncRNAs (64%) were tissue specific which agrees with previous studies showing a high proportion of tissue specificity in lncRNAs [32, 33]. In addition, several studies on lncRNAs from various vertebrate tissues [34–37] revealed that most lncRNAs in each species did not share any detectable similarity with lncRNAs in other species, suggesting rapid turnover of lncRNA repertoires. Based on these findings, it can be expected that majority of predicted lncRNAs in this study are specific to pig genome.

The 3′-RNA-seq technology sequences RNA fragments close to the 3′ end of poly-adenylated transcripts and by reducing the sequencing space/sample, provides a cheap, alternative tool to quantify gene expression level [38]. However, the accuracy of this technique in gene expression quantification is directly related to the accuracy of 3′ gene end annotation. Our Iso-seq transcripts extended the 3′ end of more than 4000 known genes in either of Ensembl or NCBI annotations. The high validation rate of these extended regions at both Ensembl and NCBI annotations using an independent 3′-RNA-seq experiment shows improvement of gene 3′-end location compared to current pig annotations. In addition, our results showed the significant effect of these 3′ end extensions on improving gene expression quantification using 3′-RNA-seq data in pig genomics.

Correct annotation of the 5' end of genes has an important role in definition of promoter proximal regulatory regions. Our novel Iso-seq-based analysis extended the 5' end of more than 3000 known genes, however the library preparation method used in this study did not specially target 5′ end caps, meaning the transcript 5′ ends are not definitive and could be truncated. A recent study [39] compared different methods required for the identification of 5′ end of transcripts and reported higher performance of the CAGE method. Coincident mapping of a large proportion of these extensions with human CAGE data aligning to the pig genome showed the improvement of current pig genome annotations at gene 5′-ends. We observed a high proportion of human CAGE reads mapping to multiple locations of the pig genome, consistent with a previous report [40]. The multiple mapping could be related to either very short human CAGE reads mapping to multigene families, species-specific copy number variants, or less likely, errors in pig genome assembly [40].

The NMD pathway protects eukaryotic cells by reducing the production of harmful truncated proteins translated from transcripts with premature termination codons [41]. NCBI (Release 109) annotation has 33 porcine transcripts annotated as NMD, while Ensembl (release 93) did not make NMD predictions in their pig genome annotation. In this study, we identified 2216 putative NMD transcript candidates related to 1710 predicted genes (7% of all predicted genes) in the porcine transcriptome for these nine tissues. Previous gene expression studies on yeast, fruitfly and human cells depleted of essential NMD factors, revealed that NMD modulates the expression of ~ 3–10% of genes [42–46]. A recent study on the chicken transcriptome using Iso-seq data [24] reported ~ 8% of predicted Iso-seq genes in this species have at least one NMD transcript.

We detected alternative splicing events in 9010 genes (37% of all genes and 90% of all genes with > 1 spliced transcript (10,064 genes)), consisting of 7525 protein-coding genes (61% of protein-coding genes) and 1485 lncRNA (12% of lncRNA genes). Similar results have been reported in pig [47] and human [48]. Also, our results revealed that alternative 3′ and 5′ splice sites and skipped exons account for the vast majority of alternative splicing events which is similar to results previously reported in other species [48, 49].

A recent study on the pig transcriptome based on PacBio Iso-seq data [28] improved previous gene structure annotation (Sscrofa10.2) in terms of novel genes (26,881) and novel transcripts (28,127). Although this study used Iso-seq data sourced from 38 porcine tissues, it has five major differences compared to our study. First, they pooled all tissue samples together prior to library creation which make it impossible to trace transcripts back to related tissues and study variability among porcine tissues. Second, sequencing depth per tissue in their experiment was lower (514,659 Iso-seq reads pooled from all 38 tissues) compared to our Iso-seq dataset (4.4 M Iso-seq reads from all nine tissues; Additional file 1: Table S1). This approach limits their datasets to capture only highly expressed genes/transcripts. Third, Illumina data used for error correction of Iso-seq reads in their study was obtained from a subset of tissues (8 tissues) with lower sequencing depth (~ 16 million reads per tissue) than we report herein (Additional file 1: Table S2). Considering the high error rate of Iso-seq data (15%) [17], this design could increase the false positive rate for novel transcript detection. Fourth, around 40% (29,992) of detected transcripts in their study (77,038) were reported to be un-spliced while this proportion was 14% (9740 transcripts) in our study. Because the authors did not describe efforts to remove genomic DNA (gDNA) contamination, the majority of these transcripts may not be real. We addressed this issue by removing predicted gDNA contamination (see Methods) and removing single-exon transcripts that had only been detected in a single tissue. High base-coverage of selected single-exon transcript by RNA-seq data implies that they are less likely to be gDNA. Fifth, a total number of 8830 loci (22% of all loci) was reported as multi-transcript genes in this study which is lower than what we obtained in our experiment (10,517 genes or 43% of all genes). This further indicates the depth of sequencing was insufficient to find lowly expressed transcripts for these genes.

## Conclusions

In-depth analysis of error-corrected long read iso-seq data for nine porcine tissues provided evidence to improve the annotation of thousands of protein-coding and lncRNA genes. These validated results increase the complexity of the predicted pig transcriptome (number of transcripts per gene, lncRNA transcripts and alternative splicing events) to that reported for the highly-annotated human genome. We provide direct evidence that the predicted novel genes and transcripts extended existing gene models, by verifying such extensions with independent ChIP-seq, 3′-RNA-seq experiment and human CAGE data. Overall, it can be concluded that the current public pig genome annotations (NCBI and Ensembl) are still far from complete and our new Iso-seq based annotation improves these annotations.

## Methods

### Sequencing the transcriptomes of nine porcine tissues by using the PacBio Iso-seq and Illumina RNA-Seq technologies

The transcriptomes of nine tissues (liver, spleen, thymus, brain, hypothalamus, diaphragm, small intestine, pituitary, longissimus muscle) from a single cross-bred pig, from which the PacBio long read-based reference genome was assembled by extraction DNA of lung tissue, was sequenced by the U.S. Meat Animal Research Center (USDA, SRA, USMARC, Clay Center, NE) using the Illumina NextSeq500 and PacBio RSII platforms for RNA-Seq and Iso-seq, respectively. Total RNA from each tissue was extracted using Trizol reagent (ThermoFisher Scientific) and the provided protocol. Briefly, approximately 100 mg of tissue was ground in a mortar and pestle cooled with liquid nitrogen, and the powder was transferred to a tube with 1 ml of Trizol reagent added and mixed by vortexing. After 5 min at room temperature, 0.2 mL of chloroform was added and the mixture was shaken for 15 s and left to stand another 3 min at room temperature. The tube was centrifuged at 12,000 x g for 15 min at 4 °C. The RNA was precipitated from the aqueous phase with 0.5 mL of isopropanol. The RNA was further purified with extended DNase I digestion to remove potential DNA contamination. The RNA quality was assessed with a Fragment Analyzer (Advanced Analytical Technologies Inc., IA). Only RNA samples of RQN above 7.0 were used for library construction. PacBio Iso-seq libraries were constructed per the PacBio Iso-seq protocol. Briefly, starting with 3 μg of total RNA, cDNA was synthesized by using SMARTer PCR cDNA Synthesis Kit (Clontech, CA) according to the Iso-seq protocol (Pacific Biosciences, CA). Then the cDNA was amplified using KAPA HiFi DNA Polymerase (KAPA Biotechnologies) for 10 or 12 cycles followed by purification and size selection into 4 fractions: 0.8–2 kb, 2–3 kb, 3–5 kb and > 5 kb. The fragment size distribution was validated on a Fragment Analyzer (Advanced Analytical Technologies Inc., IA) and quantitated on a DS-11 FX fluorometer (DeNovix, DE). After a second round of large-scale PCR amplification and end repair, SMART bell adapters were separately ligated to the cDNA fragments. Each size fraction was sequenced on 4 or 5 SMART Cells v3 using P6-C4 chemistry and 6-h movies on a PacBio RS II sequencer (Pacific Bioscience, CA). Short read RNA-Seq libraries were prepared using TruSeq stranded RNA LT kits and supplied protocol (Illumina, CA), and sequenced on a NextSeq500 platform using v2 sequencing chemistry to generate 2 × 75 paired-end reads. This published data (PRJNA351265) were used for NCBI and Ensembl gene structure annotations of the pig genome Sscrofa11.1 assembly.

### Error-correction of PacBio Iso-seq full-length cDNA reads

The Read of Insert (ROI) were determined by using ConsensusTools.sh in the SMRT-Analysis pipeline v2.0, with reads which were shorter than 300 bp and whose predicted accuracy was lower than 75% removed. Full-length, non-chimeric cDNA reads were identified by running the classify.py command. Primer sequences as well as the poly(A) tails were trimmed prior to further analysis. Paired-end Illumina RNA-Seq reads from each tissue sample were trimmed to remove the adaptor sequences and low-quality bases using Trimmomatic (v0.32) [50] with explicit option settings: ILLUMINACLIP:adapters.fa: 2:30:10:1:true LEADING:3 TRAILING:3 SLIDINGWINDOW: 4:20 LEADING:3 TRAILING:3 MINLEN:25, and overlapping paired-end reads were merged using the PEAR software (v0.9.6) [51]. Subsequently, the merged and unmerged RNA-Seq reads from the same tissue samples were in silico normalized in a mode for single-end reads by using a Trinity (v2.1.1) [21] utility, insilico_read_normalization.pl, with the following settings: --max_cov 50 --max_pct_stdev 100 --single. Errors in the full-length, non-chimeric cDNA reads were corrected with the preprocessed RNA-Seq reads from the same tissue samples by using proovread (v2.12) [17]. Untrimmed sequences with at least some regions of high accuracy in the. Trimmed.fq files were extracted based on sequence IDs in .untrimmed.fa files to balance off the contiguity and accuracy of the final reads.

### Long read transcriptome processing

The error corrected full-Length circular consensus sequences were aligned against Sscrofa11.1 pig genome assembly using GMAP (version 2017-03-17) [52] with a cut-off of 95% identity and 90% coverage. Un-spliced reads with stretch of at least 20 A’s (allowed one mismatch) in a genomic window covering 30 bp downstream of their putative terminal site were removed from analysis as they were likely gDNA contaminations. The resulted reads were collapsed and grouped into putative gene models (clustering transcripts that had at least a one nucleotide overlap) by the pbtranscript-ToFU package (https://github.com/PacificBiosciences/cDNA_primer/) with min-identity = 95%, min-coverage = 90% and max_fuzzy_junction = 5 bp, whereas the 5′-difference was not considered when collapsing the reads. The collapsed transcripts from the different tissues were then merged using in-house python scripts to create an Iso-seq based transcriptome annotation. Iso-seq transcripts were compared with annotated transcripts of Ensembl (release 93) and NCBI (Release 109) by Gffcompare [53] and transcripts were classified into 10 groups based on their exon structures (splicing junctions).

### Mapping of Illumina data

Trimmed Illumina reads were aligned against Sscrofa11.1 pig genome assembly using TopHat version 2.1.1 [54] with a cut-off of 95% identity and 90% coverage,--library-type fr-firststrand and default settings for other parameters. Quantification of transcripts was performed using Cufflinks version 2.2.1 [55] using the GTF annotation file generated by PacBio sequencing. To reduce transcription noise, single tissue detected Iso-seq transcripts were required to have minimum expression level of 0.1 FPKM (selected based on the inflection point of > 1 tissue detected Iso-seq transcripts, Fig. 22) in their detected tissue.

### ChIP-seq data analysis

Quality assurance was performed using FastQC (version 0.11.3) [https://www.bioinformatics.babraham.ac.uk/projects/fastqc/]. Adapters and low-quality bases were trimmed by running Trimmomatic (version 0.36) [50]. Trimmed reads were aligned against Sscrofa 11.1 pig genome assembly using bowtie2 [56]. Read alignment files were filtered to discard multi-mapping reads and duplicates. Model-based analysis (narrow peak model for H3K4me3 data and broad peak model for H3K36 data) of ChIP-seq (MACS 2) peak caller (version 2.1.0) [57] was used to identify regions of ChIP enrichment relative to corresponding sequenced input-DNA controls. The maximum false discovery rate of the called peaks was set to 0.05 and the data were adjusted to the size of the mappable genome size (2.5e9bp).

### 3′-RNA-seq sample preparation

Liver tissues from three healthy adult Yorkshire pigs at Iowa State University were grounded into powder in liquid nitrogen using pestle and mortar. Total RNA was extracted using the Animal Tissue RNA Purification Kit (Norgen Bioteck Corp., Thorold, ON, Canada) per the manufacturer’s instructions. The total RNA from each sample was used for stranded RNA-seq library construction separately by using the Quantseq 3′ RNA-Seq Library Prep Kit FWD for Illumina (Lexogen GmbH, Vienna, Austria). Indexed libraries for individual samples were pooled together equimolarly and sequenced using an Illumina Hiseq3000 platform to generate 50 base single end reads from ends distal to poly(A)/poly(T) ends.

### 3′-RNA-seq data analysis

Quality assurance was performed using FastQC (version 0.11.3) [http://www.bioinformatics.babraham.ac.uk/projects/fastqc/]. Adapters and low-quality bases were trimmed by running Trimmomatic (version 0.36) [50]. Trimmed reads were aligned against Sscrofa11.1 pig genome assembly using TopHat2.1.1 [54] with a cut-off of 95% identity and 90% coverage, −-library-type fr-secondststrand and default settings for other parameters. 3'RNA-seq reads uniquely mapped to pig genome were used for downstream analysis. The number of reads mapped to each gene (read counts) were calculated using HTseq version 0.10.0 [58]. Relating reads to the extended 3′ end of annotated genes was performed using bedtools [59] so that 100% of mapped 3′-RNA-seq read length was covered by the exonic region of the extended 3′ end.

### CAGE data analysis

To validate the 5′ end extension events, we used total of 45,067,042 CAP Analysis of Gene Expression (CAGE) sequences from eight matched human tissues (brain, diaphragm, liver, LD muscle pituitary, small intestine, spleen and thymus) were downloaded from FANTOM5 consortium (http://fantom.gsc.riken.jp/5/). Adapter sequences and low-quality bases were removed from the raw reads using Trimmomatic (version 0.36) [50]. Then, the trimmed reads were mapped to the Sscrofa11.1 reference genome using GMAP (version 2017-03-17) [52] with a cut-off of 95% identity and 90% coverage and --cross-species option.

### Prediction of coding and non-coding transcripts

Transcripts open reading frames (ORFs) were predicted using the stand-alone version of NCBI ORFfinder (ftp://ftp.ncbi.nlm.nih.gov/genomes/TOOLS/ORFfinder/linux-i64/) with “ATG and alternative initiation codons” as ORF start codon. The longest three ORF’s were matched to the pig, human, chicken and cow non-redundant protein sequences from NCBI (ftp://ftp.ncbi.nlm.nih.gov/blast/db) using Blastp [60] with E-value cutoff of 10^− 6^. The ORF’s with the lowest E-value to a protein were used as the representative or if no matches were found, the longest ORF was used. If the representative ORF had a stop codon that was more than 50-bp upstream of the final splice junction, it was labelled as a non-sense mediated decay transcript [24, 61]. Putative non-coding transcripts with length more than 200 bp were labelled as long non-coding RNAs [24].

### Functional enrichment analysis

The potential mechanism of action of tissue-specific genes was deciphered using ClueGO [62]. The latest update of gene ontology annotation database (GOA) [63] (January, 2019) was used in the analysis. List of genes with at least one transcript detected in a given tissue was used as background for that tissue. The GO tree interval ranged from 3 to 20 with the minimum number of genes per cluster set to three. Term enrichment was tested with a right-sided hyper-geometric test that was corrected for multiple testing by the Benjamini-Hochberg procedure [64].

## Endnotes

Mention of trade names or commercial products in this publication is solely for the purpose of providing specific information and does not imply recommendation or endorsement by the U.S. Department of Agriculture. USDA is and equal opportunity provider and employer.

## Additional file

Additional file 1: **Figure S1.** (a) Classification of biotypes for detected transcripts; length distribution of transcripts (b), exons (c), and introns (e); (e) distribution of the number of exons per transcript; (f) percentage of nucleotides at donor and acceptor sites. **Figure S2.** Percentage of PacBio transcript splice junctions supported by short-read Illumina data. **Figure S3.** Expression analysis of transcripts detected in more than one tissue by Iso-seq data. **Figure S4.** Number of PacBio transcripts detected in each tissue and their intersections with other tissues using UpSetR [1]. Blue color identifies the proportion of single tissue detected transcripts by PacBio data that were also detected by Illumina reads in at least one other tissue (see the text for more details). **Figure S5.** (a) Distribution of class “k” transcripts (contains reference) across Ensembl and NCBI annotations, (b) biotypes of transcripts with “k” structure in both Ensembl and NCBI annotations. (c) Expression level of class “k” transcripts across tissues. (d) Classification of class “k” transcripts based on the number of tissues in which they were detected. **Figure S6.** Biotypes of different transcript types based on Ensembl (a) and NCBI (b) annotations. **Figure S7.** Classification of class “s” transcripts based on the number of tissues in which they were detected. **Figure S8.** Example of validation of novel intergenic Iso-seq gene using matched RNA-seq reads and independent liver ChIP-seq (H3K4me3 and H3K36me3) and 3′-RNA-seq experiments. **Figure S9.** Venn diagram of the number of livers detected Ensembl (a) and NCBI (b) genes with validated extended 3′ end across different samples of an independent liver 3′-RNA-seq experiment. **Figure S10.** Example of validation of extended 3′ annotation using an independent liver 3′-RNA-seq experiment. **Figure S11**. Effect of extended annotation on the expression level of Ensembl genes using liver 3′-RNA-seq reads. Genes with same expression in both Iso-seq and Ensembl annotations were marked with red color. Blue line in each graph shows the average of Iso-seq gene expression fold changes over of their matched Ensembl genes in log2 scale that is equal to 0.485 or 40% expression increase. **Figure S12.** (a) Definition of 5′ candidate region and (b) number of genes with validated candidate 5′ end across different annotations. **Figure S13.** Example of validation of extended 5′ annotation using an independent Human CAGE data. **Table S1.** PacBio Iso-seq sequence alignment statistics. **Table S2.** Illumina sequence alignment statistics. **Table S3.** Mapping statistics and quality metrics used for the evaluation of ChIP-seq experiment. **Table S4.** 3′-RNA-seq sequences alignment statistics. **Table S5.** Functional enrichment analysis of tissue-specific (TS) genes in different porcine tissues. (DOCX 97254 kb)

## Abbreviations

A3Es: Alternative 3′ splice site exons
A5Es: alternative 5′ splice site exons
bp: base pairs
CAGE: CAP Analysis of Gene Expression
ChIP-seq: Chromatin immunoprecipitation sequencing
EBI: European Bioinformatics Institute
Ensembl specific Iso-seq genes: Iso-seq genes were found in the Ensembl gene set, by not in the NCBI gene set
ESTs: Expressed Sequence Tags
gDNA: genomic DNA
GO: Gene Ontology
H3K36me3: tri-methylation of lysine 36 on histone H3
H3K4me3: tri-methylation of lysine 4 on histone H3
Iso-seq: single-molecule long-read isoform sequencing
LD: longissimus dorsi
lncRNA: long non-coding RNA
NCBI specific Iso-seq genes: Iso-seq genes that were found in the NCBI gene set but not in the Ensembl annotation gene set
NCBI: National Center for biotechnology Information
NGS: Next-generation sequencing
NMD: non-sense mediated decay
nt: nucleotides
ORFs: open reading frames
PacBio: Pacific Biosciences
RNA-seq: Illumina RNA sequencing
ROI: Read of Insert
SE: skipped exon
*Sus scrofa domesticus*: Domestic pigs
tpg: transcripts per gene
UTRs: Untranslated regions
