# Supplementary material for: Improved annotation of the domestic pig genome through integration of Iso-Seq and RNA-seq data
Source: BMC Genomics. 2019 May 7;20:344. doi: 10.1186/s12864-019-5709-y (PMC6505119; doi:10.1186/s12864-019-5709-y)
Supplement: Supplementary file 1 — Figure S1. (a) Classification of biotypes for detected transcripts; length distribution of transcripts (b), exons (c), and introns (e); (e) distribution of the number of exons per transcript; (f) percentage of nucleotides at donor and acceptor sites. Figure S2. Percentage of PacBio transcript splice junctions supported by short-read Illumina data. Figure S3. Expression analysis of transcripts detected in more than one tissue by Iso-seq data. Figure S4. Number of PacBio transcripts detected in each tissue and their intersections with other tissues using UpSetR [1]. Blue color identifies the proportion of single tissue detected transcripts by PacBio data that were also detected by Illumina reads in at least one other tissue (see the text for more details). Figure S5. (a) Distribution of class “k” transcripts (contains reference) across Ensembl and NCBI annotations, (b) biotypes of transcripts with “k” structure in both Ensembl and NCBI annotations. (c) Expression level of class “k” transcripts across tissues. (d) Classification of class “k” transcripts based on the number of tissues in which they were detected. Figure S6. Biotypes of different transcript types based on Ensembl (a) and NCBI (b) annotations. Figure S7. Classification of class “s” transcripts based on the number of tissues in which they were detected. Figure S8. Example of validation of novel intergenic Iso-seq gene using matched RNA-seq reads and independent liver ChIP-seq (H3K4me3 and H3K36me3) and 3′-RNA-seq experiments. Figure S9. Venn diagram of the number of livers detected Ensembl (a) and NCBI (b) genes with validated extended 3′ end across different samples of an independent liver 3′-RNA-seq experiment. Figure S10. Example of validation of extended 3′ annotation using an independent liver 3′-RNA-seq experiment. Figure S11. Effect of extended annotation on the expression level of Ensembl genes using liver 3′-RNA-seq reads. Genes with same expression in both Iso-seq and Ensembl annotations were mar [file 12864_2019_5709_MOESM1_ESM.docx]

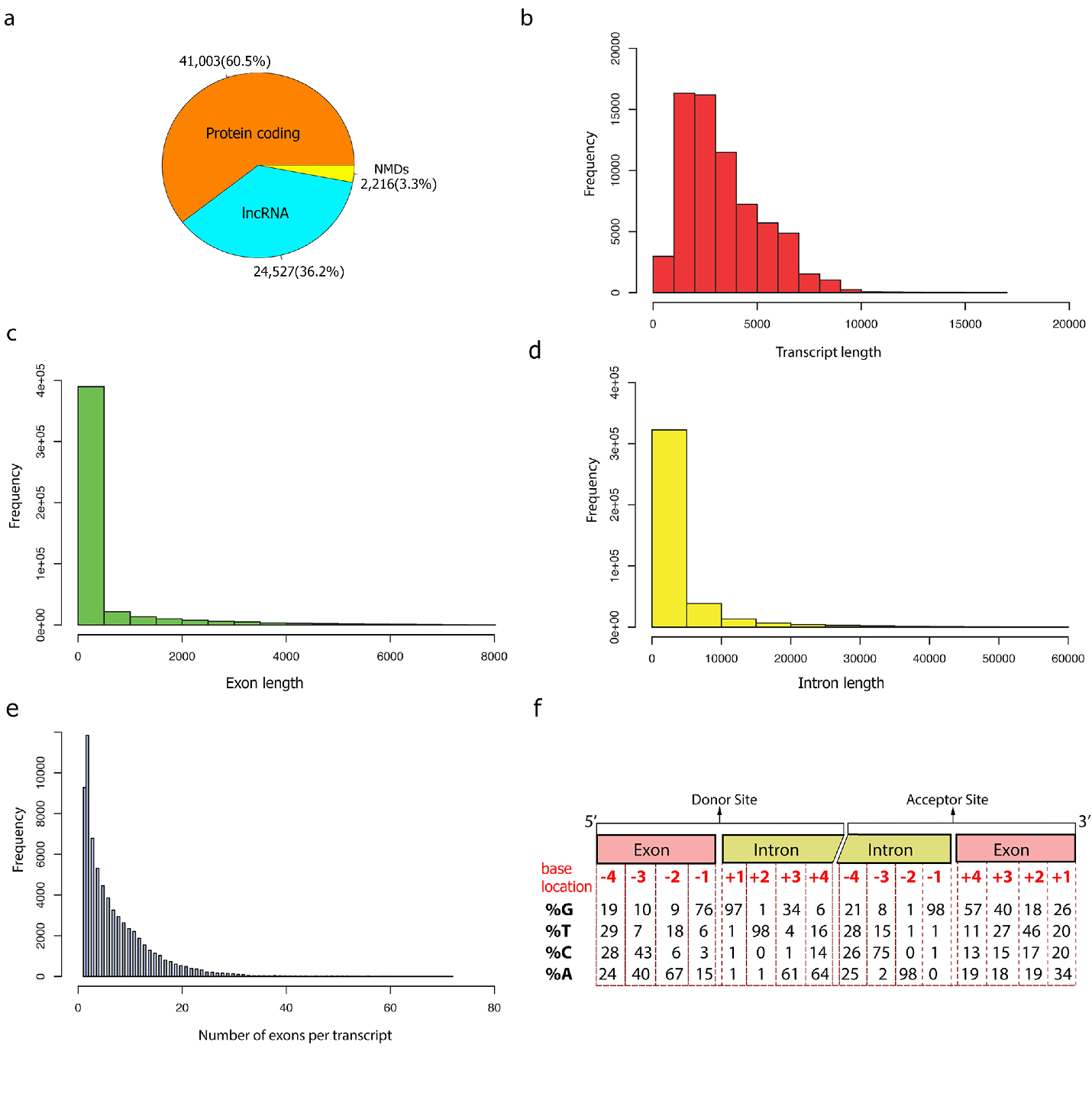


**Figure S1.** (a) Classification of biotypes for detected transcripts; length distribution of transcripts (b), exons (c), and introns (e); (e) distribution of the number of exons per transcript; (f) percentage of nucleotides at donor and acceptor sites


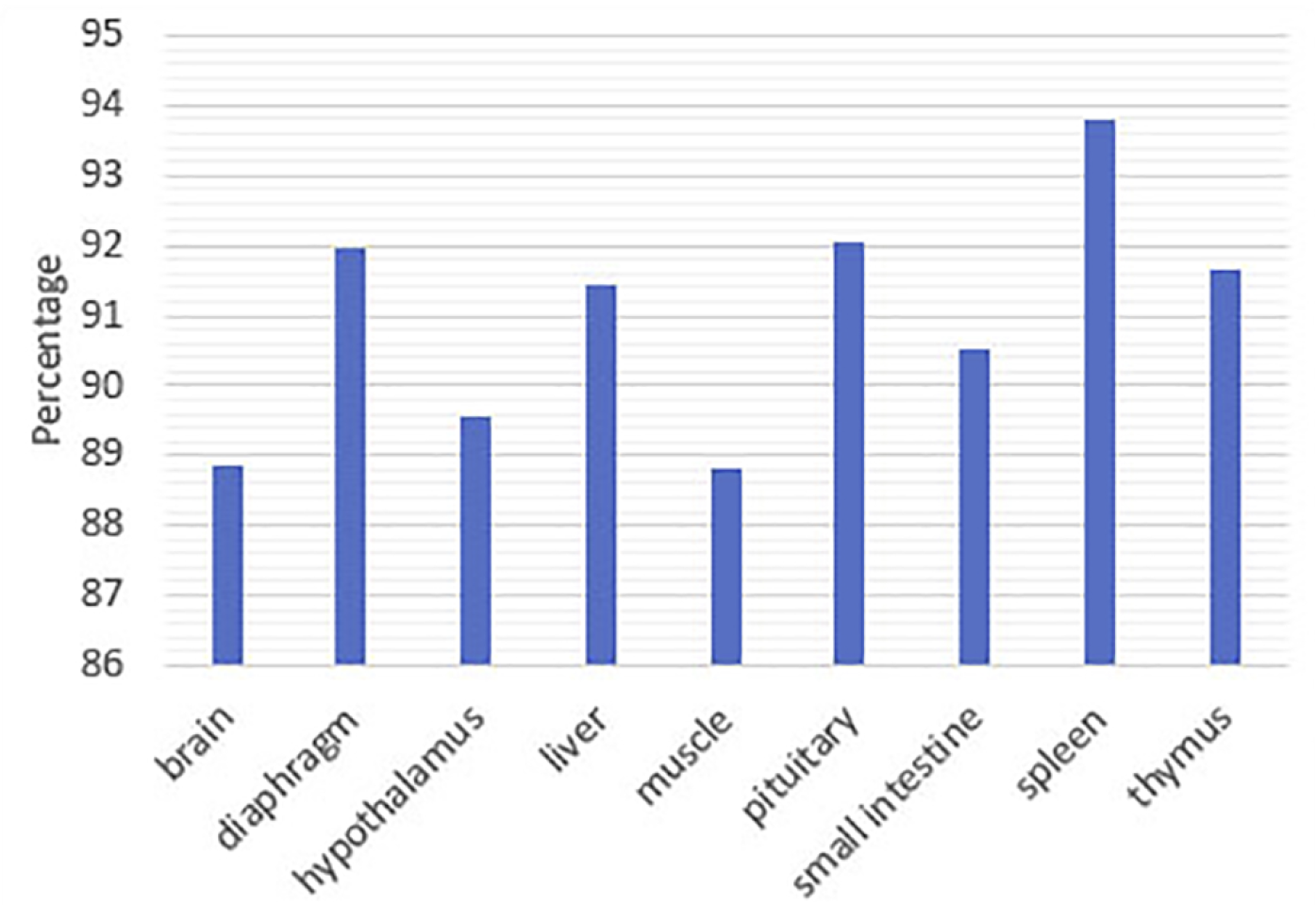


**Figure S2.** Percentage of PacBio transcript splice junctions supported by short-read Illumina data.


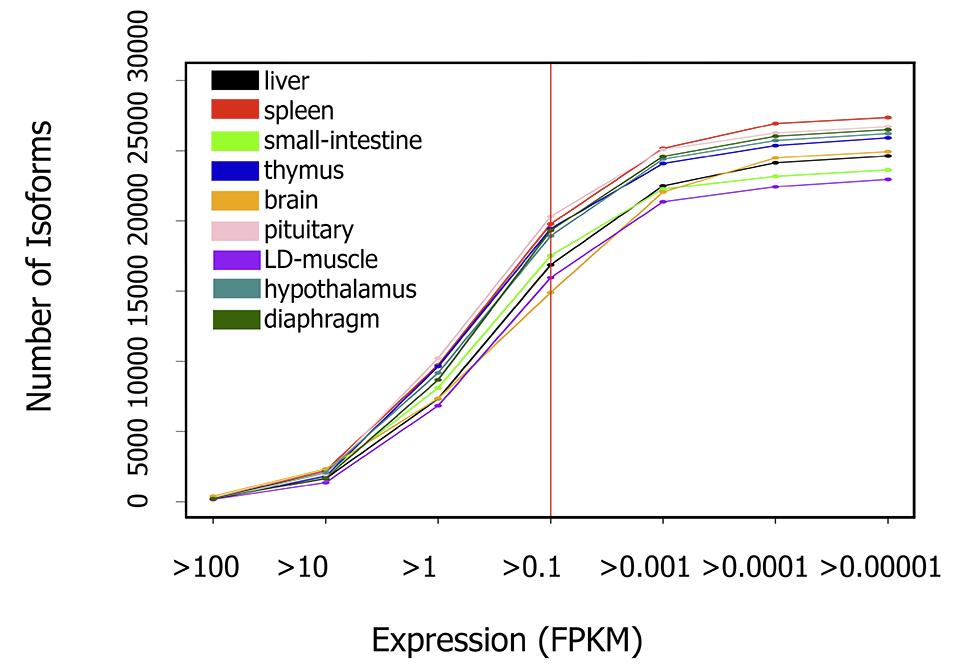


**Figure S3.** Expression analysis of transcripts detected in more than one tissue by Iso-seq data


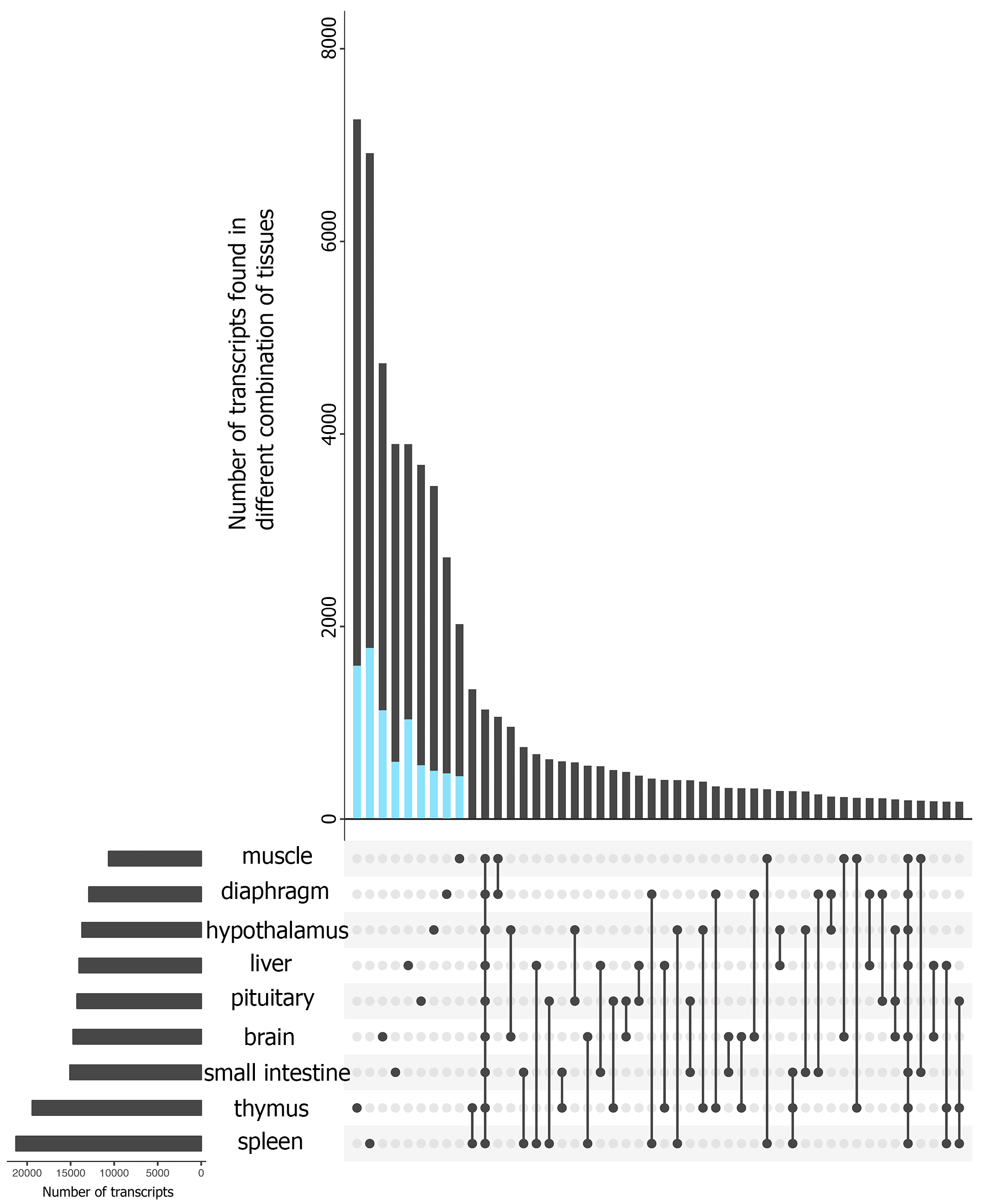


**Figure S4.** Number of PacBio transcripts detected in each tissue and their intersections with other tissues using UpSetR [1]. Blue color identifies the proportion of single tissue detected transcripts by PacBio data that were also detected by Illumina reads in at least one other tissue (see the text for more details).


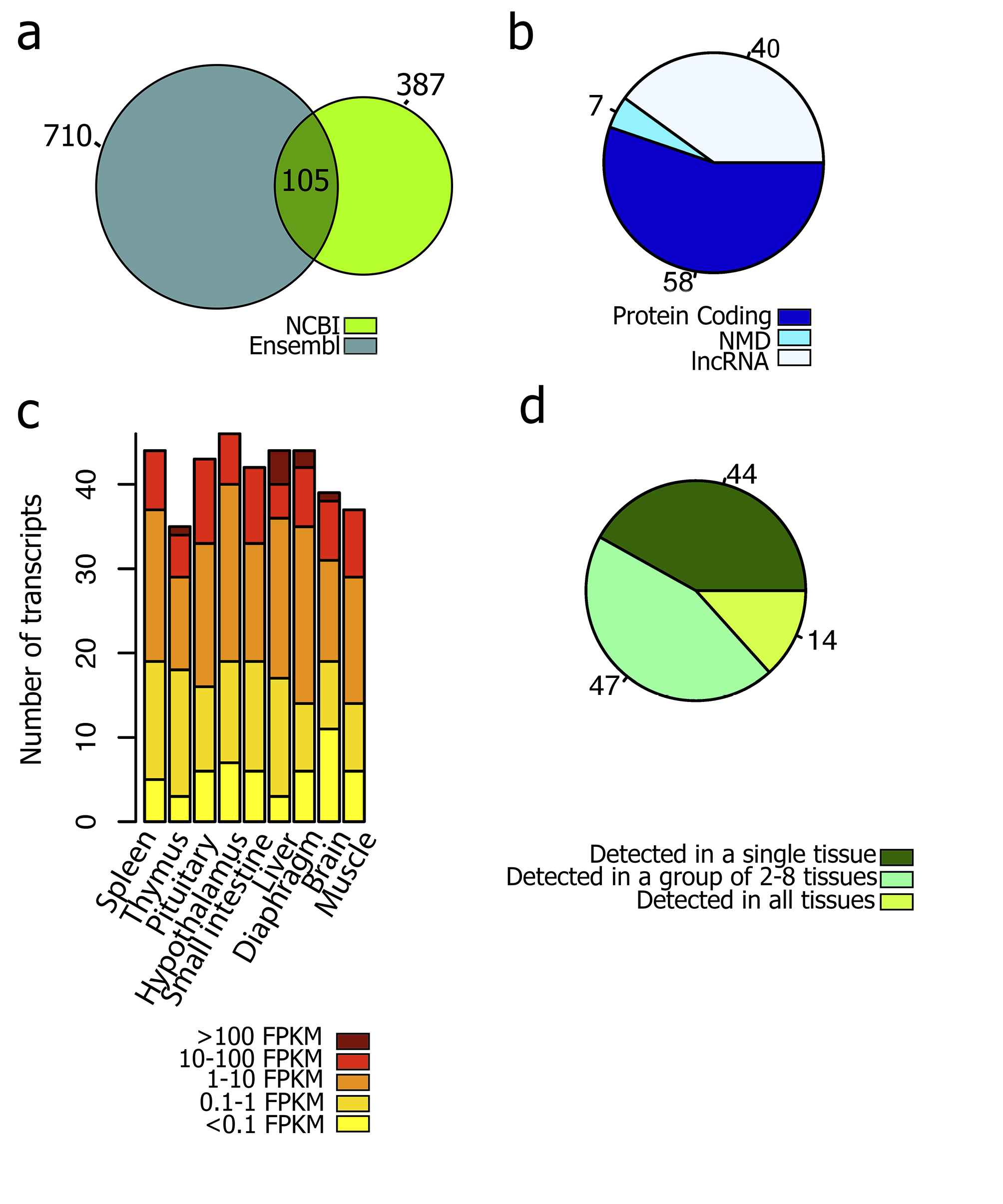


**Figure S5.** (a) Distribution of class “k” transcripts (contains reference) across Ensembl and NCBI annotations, (b) biotypes of transcripts with “k” structure in both Ensembl and NCBI annotations. (c) Expression level of class “k” transcripts across tissues. (d) Classification of class “k” transcripts based on the number of tissues in which they were detected.


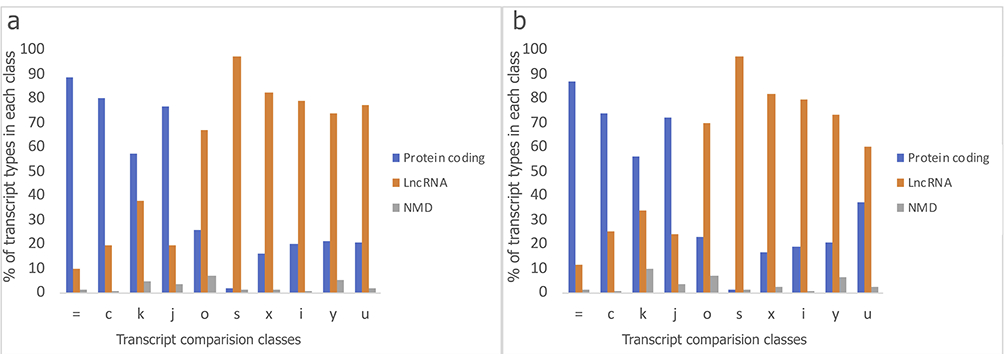


**Figure S6.** Biotypes of different transcript types based on Ensembl (a) and NCBI (b) annotations.


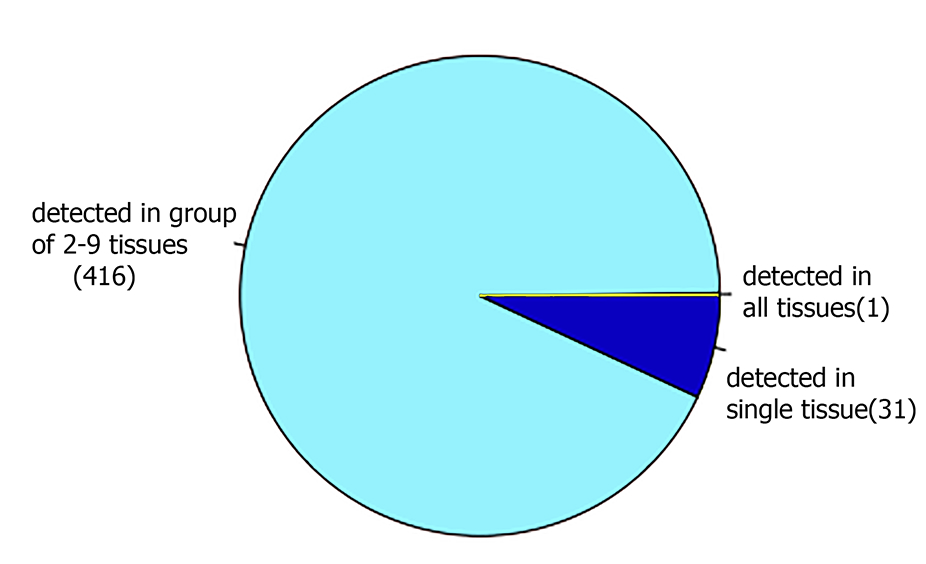


**Figure S7.** Classification of class “s” transcripts based on the number of tissues in which they were detected.


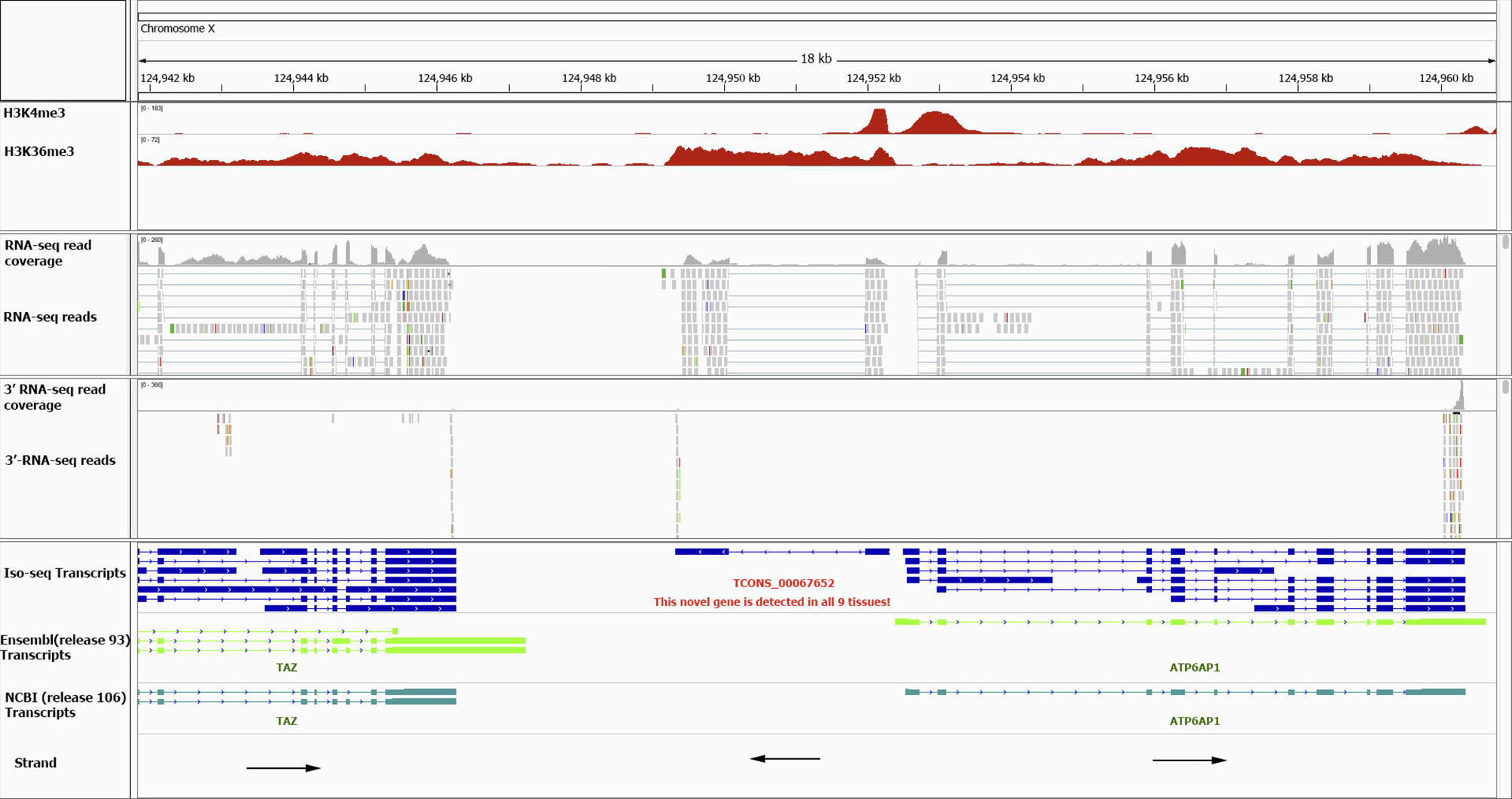


**Figure S8.** Example of validation of novel intergenic Iso-seq gene using matched RNA-seq reads and independent liver ChIP-seq (H3K4me3 and H3K36me3) and 3’-RNA-seq experiments.


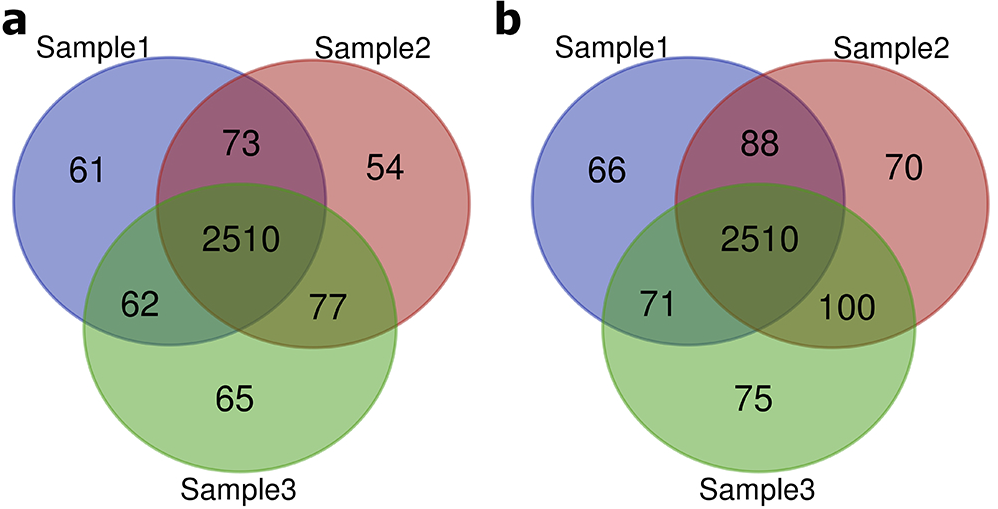


**Figure S9.** Venn diagram of the number of livers detected Ensembl (a) and NCBI (b) genes with validated extended 3’ end across different samples of an independent liver 3’-RNA-seq experiment.


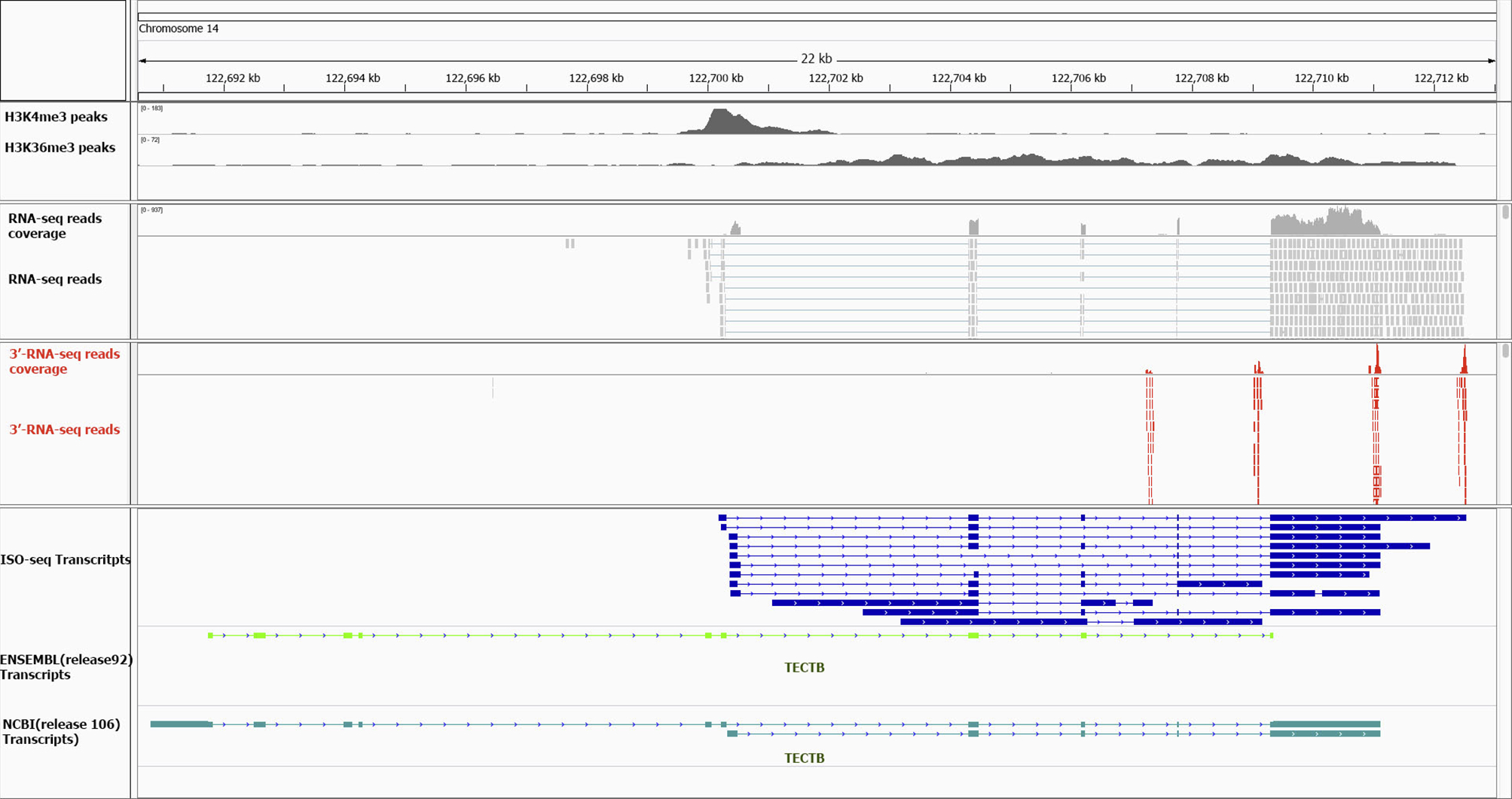


**Figure S10.** Example of validation of extended 3’ annotation using an independent liver 3’-RNA-seq experiment.


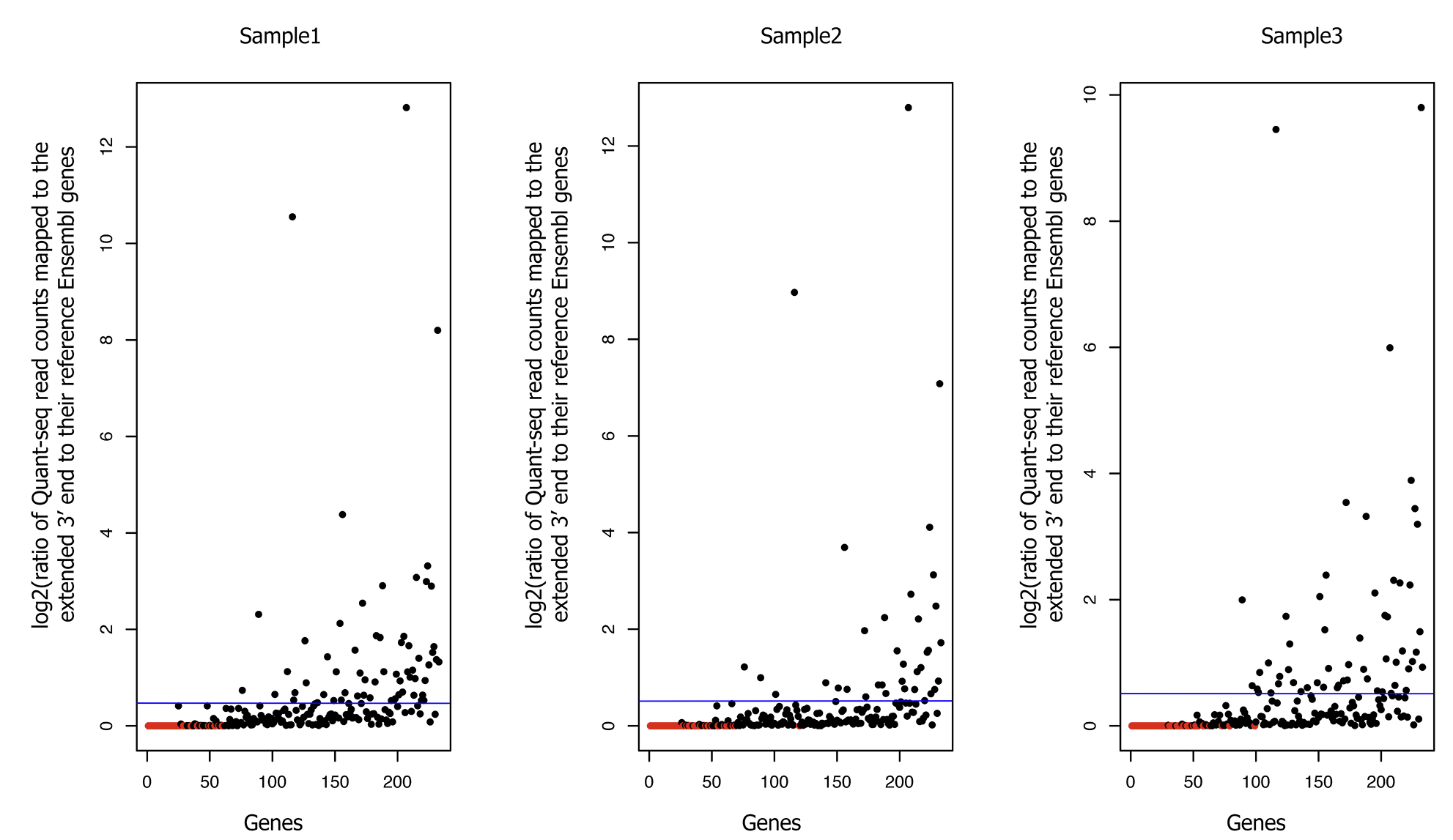


**Figure S11**. Effect of extended annotation on the expression level of Ensembl genes using liver 3’-RNA-seq reads. Genes with same expression in both Iso-seq and Ensembl annotations were marked with red color. Blue line in each graph shows the average of Iso-seq gene expression fold changes over of their matched Ensembl genes in log2 scale that is equal to 0.485 or 40% expression increase.


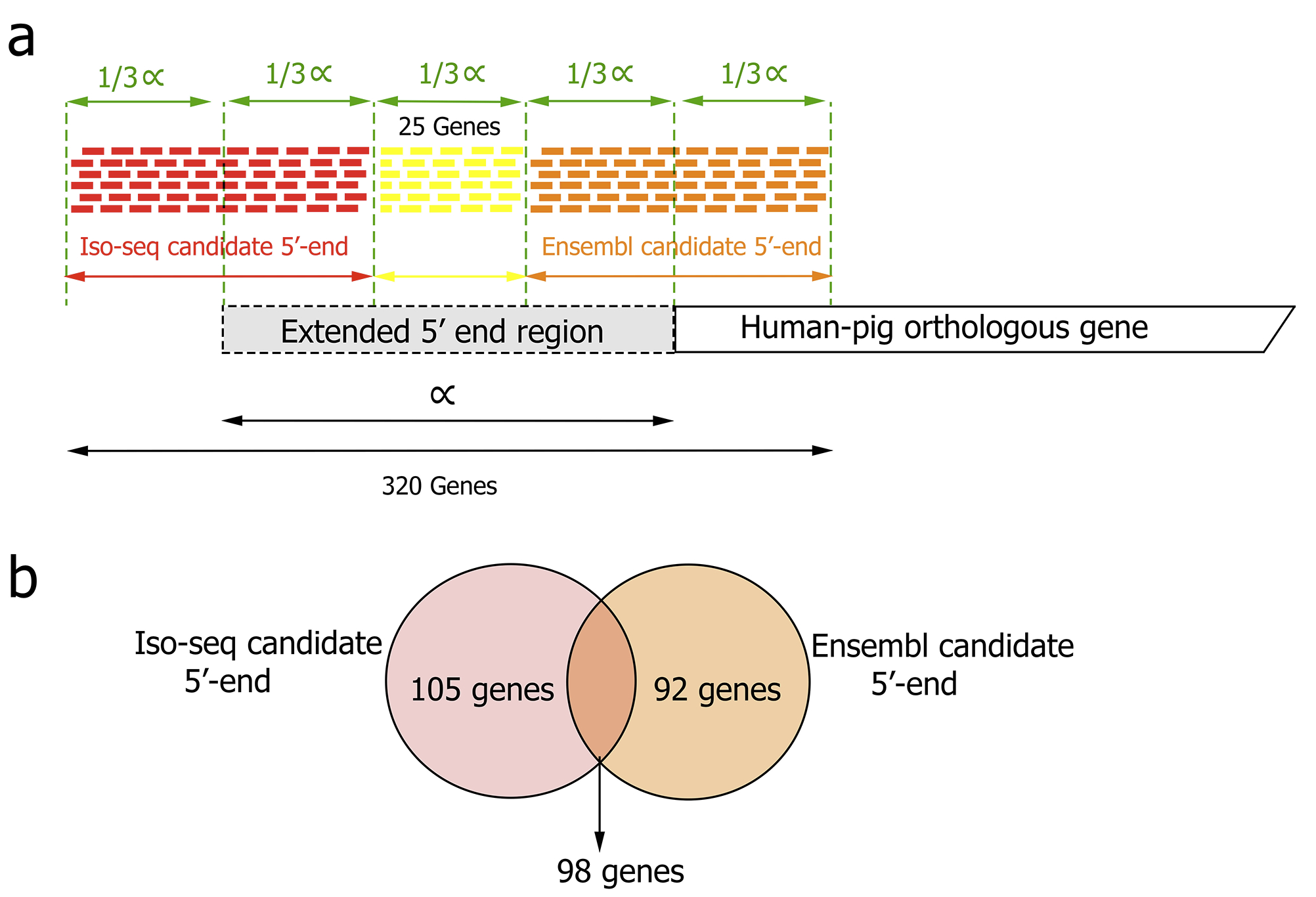


**Figure S12.** (a) Definition of 5’ candidate region and (b) number of genes with validated candidate 5’ end across different annotations.


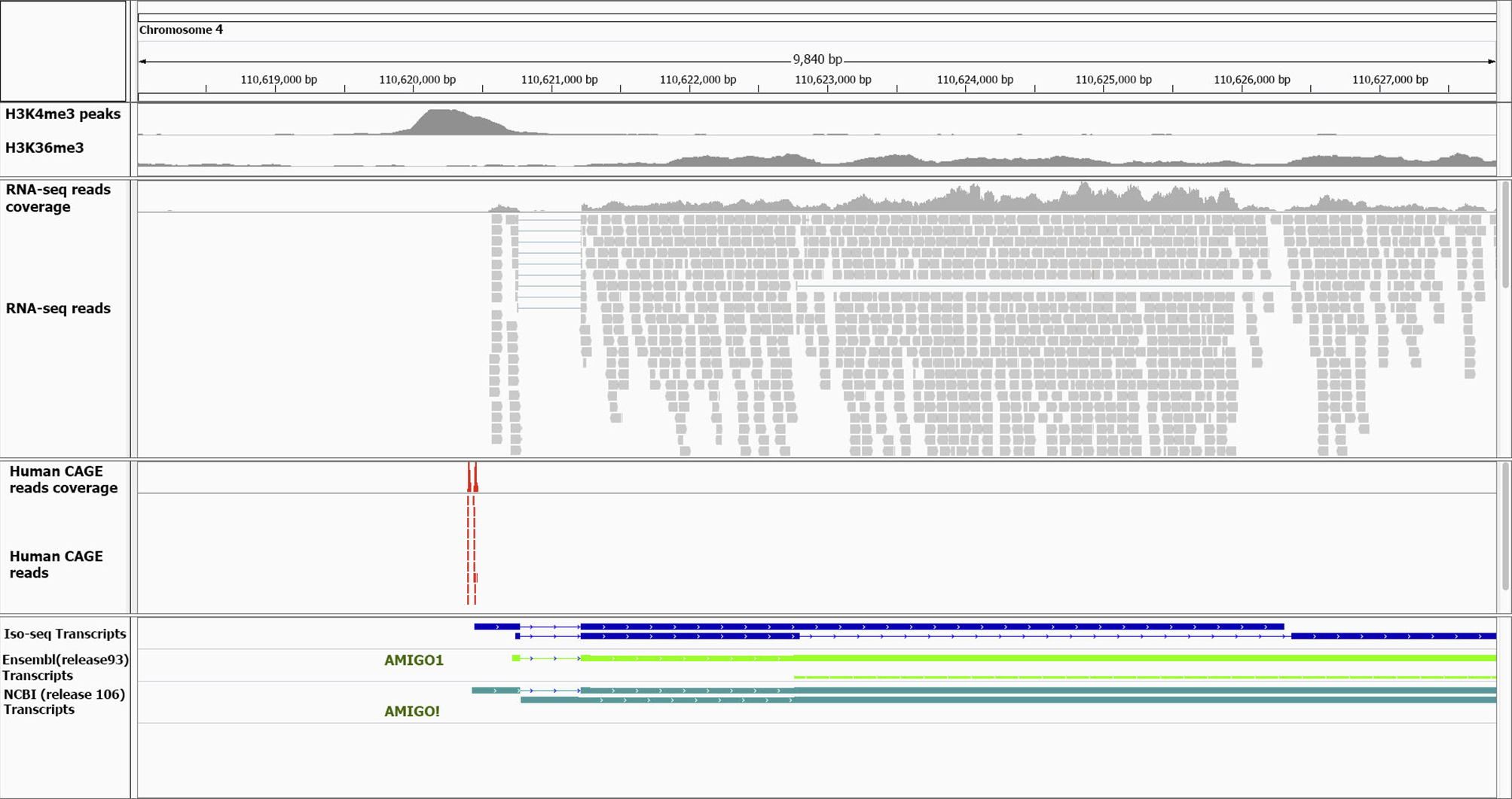


**Figure S13.** Example of validation of extended 5’ annotation using an independent Human CAGE data.

| **Table S1.** PacBio Iso-seq sequence alignment statistics | | | | |
| --- | --- | --- | --- | --- |
| Tissue | Total sequences | Mapped sequences | Uniquely mapped sequences | Multi-mapped sequences |
| Brain | 398,629 | 390,958(98%) | 357,662(92%) | 33,296(8%) |
| Diaphragm | 459,911 | 452,644(98%) | 418,317(92%) | 34,327(8%) |
| Hypothalamus | 414,021 | 404,112(97%) | 348,526(86%) | 55,586(14%) |
| Muscle | 410,420 | 405,636(98%) | 377,247(93%) | 28,389(7%) |
| Liver | 588,957 | 581,572(99%) | 541,553(93%) | 40,019(7%) |
| Pituitary | 411,562 | 402,195(98%) | 345,370(86%) | 56,825(14%) |
| Small-intestine | 494,538 | 487,671(99%) | 443,970(91%) | 43,701(9%) |
| Spleen | 674,053 | 663,201(98%) | 605,765(91%) | 57,436(9%) |
| Thymus | 567,700 | 556,801(98%) | 489,629(88%) | 67,172(12%) |

| **Table S2.** Illumina sequence alignment statistics | | | | |
| --- | --- | --- | --- | --- |
| Tissue | Initial sequences | Mapped sequences | Uniquely mapped sequences | Multi-mapped sequences |
| Brain | 104,792,366 | 100,584,570(94%) | 94,390,747(94%) | 6,193,823(6%) |
| Diaphragm | 51,603,793 | 49,152,714(95%) | 47,891,573(97%) | 1,261,141(3%) |
| Hypothalamus | 54,392,874 | 52,566,220(96%) | 51,790,383(98%) | 775,837(2%) |
| Muscle | 39,658,838 | 38,496,157(97%) | 37,311,751(97%) | 1,184,406(3%) |
| Liver | 60,566,794 | 57,497,615(94%) | 56,025,172(97%) | 1,472,443(3%) |
| Pituitary | 39,475,285 | 37,746,041(95%) | 36,896,986(98%) | 849,055(2%) |
| Small-intestine | 32,689,730 | 31,334,659(95%) | 30,346,773(97%) | 987,886(3%) |
| Spleen | 69,207,878 | 65,018,175(93%) | 62,720,649(96%) | 2,297,526(4%) |
| Thymus | 47,233,339 | 45,370,171(95%) | 44,618,283(98%) | 751,888(2%) |

| **Table S3.** Mapping statistics and quality metrics used for the evaluation of ChIP-seq experiment. | | | | | | | |
| --- | --- | --- | --- | --- | --- | --- | --- |
| Sample | Total # of reads | %of uniquely mapped reads | #Of nun-redundant reads | NRF^1^ | PBC^2^ | Complexity^3^ | Peak enrichment^4^ |
| S1-input | 106,152,934 | 79.03 | 90,612,898 | 0.86 | 0.90 | Compliant | - |
| S1-K36 | 99,207,846 | 80.91 | 80,657,703 | 0.87 | 0.87 | Compliant | 62.30% |
| S1-K4 | 128309209 | 87.80 | 78,288,726 | 0.89 | 0.74 | Acceptable | 43.74% |
| S2-input | 78,082,658 | 79.11 | 67,456,744 | 0.86 | 0.92 | Compliant | - |
| S2-K36 | 110,771,101 | 80.61 | 88,739,937 | 0.87 | 0.86 | Compliant | 55.59% |
| S2-K4 | 75,833,210 | 87.48 | 45,606,108 | 0.89 | 0.77 | Acceptable | 45.39% |
| S3-input | 106211340 | 78.21 | 91,283,383 | 0.86 | 0.92 | Compliant | - |
| S3-K36 | 110,029,483 | 80.31 | 90,937,523 | 0.87 | 0.88 | Compliant | 63.90% |
| S3-K4 | 78,307,935 | 83.99 | 55,966,614 | 0.88 | 0.80 | Compliant | 31.52% |
| S4-input | 110,344,003 | 78.94 | 93,958,151 | 0.86 | 0.91 | Compliant | - |
| S4-K36 | 81,969,506 | 80.80 | 68,274,809 | 0.87 | 0.88 | Compliant | 67.22% |
| S4-K4 | 109,079,063 | 86.82 | 70,418,551 | 0.88 | 0.76 | Acceptable | 41.34% |
| ^1^Non-Redundant Fraction: Number of distinct uniquely mapping reads/ Total number of reads [2].  ^2^Number of genomic locations where exactly one read maps uniquely/ number of distinct genomic locations to which some read maps uniquely [2].  ^3^Library complexities were identified using PBC and NRF based on ENCODE definition (<https://www.encodeproject.org/data-standards/terms/>)  ^4^The number of reads that reside in the peak regions. In general, enrichment values <1% may indicate a poor ChIP experiment [2]. | | | | | | | |

| **Table S4.** 3’-RNA-seq sequences alignment statistics | | | | |
| --- | --- | --- | --- | --- |
| Sample | Initial sequences | Mapped sequences | Uniquely mapped sequences | Multi-mapped sequences |
| Sample1 | 15,301,072 | 14,348,750(94%) | 12,032,945(84%) | 2,315,805(16%) |
| Sample2 | 10,589,121 | 9,772,957(92%) | 8,562,248(88%) | 1,210,709(12%) |
| Sample3 | 13,472,295 | 12,483,660(93%) | 10,888,477(87%) | 1,595,183(13%) |

| **Table S5.** Functional enrichment analysis of tissue-specific (TS) genes in different porcine tissues. | | | | |
| --- | --- | --- | --- | --- |
| Tissue | Number of TS genes^1^ | GO term^2^ | Genes in GO term^3^ | p-value^4^ |
| Thymus | 1,196 | protein prenylation^5^ | 3/9 | 5.0E-3 |
|  |  | DNA replication^6^ | 9/180 | 5.2E-3 |
|  |  | protein heterooligomerization^7^ | 4/48 | 7.7E-3 |
| Brain | 836 | regulation of synapse assembly | 11/45 | 1.3E-5 |
|  |  | nervous system development | 46/731 | 3.7E-5 |
|  |  | regulation of nervous system development | 25/303 | 1.4E-4 |
| Spleen | 794 | G protein-coupled receptor signaling pathway^5^ | 20/224 | 2.5E-3 |
|  |  | adenylate cyclase-activating G protein-coupled receptor signaling pathway | 8/46 | 7.8E-3 |
|  |  | adaptive immune response | 14/158 | 1.3E-2 |
| Small intestine | 613 | intestinal absorption | 3/34 | 1.1E-2 |
|  |  | phospholipid catabolic process | 3/30 | 1.1E-2 |
|  |  | digestive system process | 5/86 | 2.0E-2 |
| Pituitary | 494 | endocrine system development | 5/43 | 1.3E-2 |
|  |  | central nervous system development | 13/273 | 1.4E-2 |
|  |  | regulation of peptide hormone secretion | 5/46 | 1.4E-2 |
| Liver | 484 | organic acid metabolic process | 26/418 | 6.8E-5 |
|  |  | protein processing | 11/106 | 5.3E-4 |
|  |  | protein maturation | 12/142 | 6.8E-4 |
| Hypothalamus | 454 | central nervous system neuron differentiation | 7/163 | 1.4E-3 |
|  |  | regulation of neurotransmitter transport | 5/94 | 1.9E-3 |
|  |  | synaptic transmission, dopaminergic | 3/28 | 2.7E-3 |
| Diaphragm | 249 | sarcomere organization | 3/29 | 1.1E-2 |
|  |  | actin-mediated cell contraction | 3/29 | 1.3E-2 |
|  |  | actin filament-based movement | 3/42 | 1.6E-2 |
| Muscle | 197 | positive regulation of mitotic nuclear division | 3/25 | 3.3E-3 |
|  |  | regulation of nuclear division | 4/89 | 8.0E-3 |
|  |  | positive regulation of mitotic cell cycle | 3/71 | 4.5E-2 |
| ^1^ NCBI gene set (release 109) was used to annotate predicted Iso-seq genes as it’s more concordant with our new Iso-seq based pig gene set annotation than Ensembl gene set (release 93) (Fig. 4a). | | | | |
| ^2^ Top three over-represented biological process Gene Ontology (GO) terms were reported in each tissue. | | | | |
| ^3^ The two values listed in this column refer to the number of genes associated with the over-represented GO term in TS genes and the number of genes associated with the same GO term in pig genome. | | | | |
| ^4^ The P-values were adjusted using the Benjamini-Hochberg procedure [3] | | | | |
| ^5^ Recent study on human thymus tissue showed indispensable role of protein prenylation in T-cell survival [4]. | | | | |
| ^6^ The importance of cell-division in regulation of immune system process has been shown in several studies [5-7] | | | | |
| ^7^ Protein heterooligomerization is a key element in detection of pathogen-associated molecular patterns by cellular pattern recognition receptors and in immune signaling [8, 9]. | | | | |

**References**

1. Conway JR, Lex A, Gehlenborg N: UpSetR: an R package for the visualization of intersecting sets and their properties. *Bioinformatics* 2017, 33:2938-2940.

2. Bailey T, Krajewski P, Ladunga I, Lefebvre C, Li Q, Liu T, Madrigal P, Taslim C, Zhang J: Practical guidelines for the comprehensive analysis of ChIP-seq data. *PLoS Comput Biol* 2013, 9:e1003326.

3. Kim KI, van de Wiel MA: Effects of dependence in high-dimensional multiple testing problems. *BMC Bioinformatics* 2008, 9:114.

4. Lacher SM, Bruttger J, Kalt B, Berthelet J, Rajalingam K, Wortge S, Waisman A: HMG-CoA reductase promotes protein prenylation and therefore is indispensible for T-cell survival. *Cell Death Dis* 2017, 8:e2824.

5. Kan A, Hodgkin PD: Mechanisms of cell division as regulators of acute immune response. *Syst Synth Biol* 2014, 8:215-221.

6. Hawkins ED, Turner ML, Wellard CJ, Zhou JH, Dowling MR, Hodgkin PD: Quantal and graded stimulation of B lymphocytes as alternative strategies for regulating adaptive immune responses. *Nat Commun* 2013, 4:2406.

7. Hawkins ED, Turner ML, Dowling MR, van Gend C, Hodgkin PD: A model of immune regulation as a consequence of randomized lymphocyte division and death times. *Proc Natl Acad Sci U S A* 2007, 104:5032-5037.

8. Zhao J, Li J, Xu S, Feng P: Emerging Roles of Protein Deamidation in Innate Immune Signaling. *J Virol* 2016, 90:4262-4268.

9. Wu B, Peisley A, Tetrault D, Li Z, Egelman EH, Magor KE, Walz T, Penczek PA, Hur S: Molecular imprinting as a signal-activation mechanism of the viral RNA sensor RIG-I. *Mol Cell* 2014, 55:511-523.
